# A parameter estimation method for fluorescence lifetime data

**DOI:** 10.1186/s13104-015-1176-y

**Published:** 2015-06-09

**Authors:** Daniel Sewell, Hajin Kim, Taekjip Ha, Ping Ma

**Affiliations:** Department of Statistics, University of Illinois Urbana-Champaign, 725 S. Wright St., Champaign, IL 61820 USA; School of Life Sciences, Ulsan National Institute of Science and Technology, Ulsan, Republic of Korea; Center for Soft and Living Matter, Institute for Basic Science, Ulsan, Republic of Korea; Department of Physics, University of Illinois Urbana-Champaign, 1110 W. Green St., 61801 Urbana, IL USA; Department of Statistics, University of Georgia, 101 Cedar Street, Athens, GA 30602 USA

**Keywords:** Single-molecule fluorescence lifetime, Numerical optimization, Overfitting data, Mixture distribution

## Abstract

**Background:**

When modeling single-molecule fluorescence lifetime experimental data, the analysis often involves fitting a biexponential distribution to binned data. When dealing with small sample sizes, there is the potential for convergence failure in numerical optimization, for convergence to local optima resulting in physically unreasonable parameter estimates, and also for overfitting the data.

**Results:**

To avoid the problems that arise in small sample sizes, we have developed a gamma conversion method to estimate the lifetime components. The key idea is to use a gamma distribution for initial numerical optimization and then convert the gamma parameters to biexponential ones via moment matching. A simulation study is undertaken with 30 unique configurations of parameter values. We also performed the same analysis on data obtained from a fluorescence lifetime experiment using the fluorophore Cy3. In both the simulation study and the real data analysis, fitting the biexponential directly led to a large number of data sets whose estimates were physically unreasonable, while using the gamma conversion yielded estimates consistently close to the true values.

**Conclusions:**

Our analysis shows that using numerical optimization methods to fit the biexponential distribution directly can lead to failure to converge, convergence to physically unreasonable parameter estimates, and overfitting the data. The proposed gamma conversion method avoids these numerical difficulties, yielding better results.

**Electronic supplementary material:**

The online version of this article (doi:10.1186/s13104-015-1176-y) contains supplementary material, which is available to authorized users.

## Findings

### Background

In the single-molecule fluorescence lifetime experiments, a fluorophore is attached to the molecule under study, which is placed in a focal volume illuminated by a pulsed laser. The fluorophore emits photons when excited by the pulsed laser. The time length that it takes for the fluorophore to release the photon from the moment that it is excited is termed as the photon delay time (or fluorescence lifetime). The photon delay time is recorded by time-correlated single photon counting device [1].

Because the dye’s photon emission pattern depends on its photophysical state and molecular environment which are then affected by the conformational or electronic state of the molecule with which it is interacting (e.g., the active and inactive states of an enzyme could have different effect on the dye’s photon emission intensity in certain cases), by examining how the photon emission pattern fluctuates over time, one can investigate the underlying dynamics of the molecules. It is thus of interest to study the photon delay time.

This time lapse (or fluorescence lifetime) data are often binned to form count data. The decay curve describing the stochasticity of the continuous time lapse is then indirectly estimated from the count data. This leads to a two-level hierarchical model, where the first level models the binned counts and the second level models the continuous time lapse. That is, the stochasticity of the count data is determined by certain bin probabilities (the first level), and these probabilities are in turn modeled by the decay curve corresponding to the time lapse (the second level).

More specifically, conditioning on the total number of photons counted, the bin counts follow a multinomial distribution. The probability that a photon is counted during a given time interval (bin) is determined by the cumulative distribution function (cdf) of the time lapse. A mixture of exponential probability density functions (pdf) is most widely used to model the decay curve of the fluorescence lifetime [2–4]. The specific context considered here is that the data follow a two-component mixture of exponentials (biexponential distribution). Furthermore, we assume that, by carefully controlled experimental conditions, the major lifetime component is known (though as we will see later, this restriction is not necessary to our method of parameter estimation) and we aim to estimate the second component.

Parameter estimation in this context can often be difficult, unreliable and biased. Novikov et al. [5] showed that the parameter estimation for biexponential decays is more critical and depends on the detection procedure, leaving substantial obscurity on the estimation. Early work to address this was done by Sasaki and Masuhara [6], who used a convolved autoregressive model that can be fitted using the least squares (LS) method. This approach was made more efficient by Enderlein and Erdmann [7]. However, employing LS leads to unnecessary bias [8].

A better statistical approach would be to try to find the maximum likelihood estimators (MLEs) of the biexponential distribution involved in this hierarchical model. Indeed, using LS is equivalent to finding the MLE while assuming the bin counts follow a normal pdf; however, this assumption of normality is clearly not the case, as small bin counts and sparsity of the data make the normal model an inadequate approximation of the distribution of the bin counts [9]. The fact that finding the MLE is more appropriate than using LS has been reviewed by Maus et al. [10], Edel et al. [11], and Laurence and Chromy [9].

When dealing with mixture models such as a biexponential pdf, the expectation–maximization (EM) algorithm has been widely used for finding MLE’s [12]. In this hierarchical setting, however, the EM algorithm may be both difficult to implement and slow to converge, and hence other numerical optimization methods may be employed. With a small sample size and small bin width, there will inevitably be a zero count in many of the bins [8], and such sparsity of the data may cause these numerical optimization techniques to be unstable and error-prone in finding the MLE for a mixture distribution. The commonly used direct search Nelder–Mead algorithm [13] was found to perform poorly with such a two level hierarchical model (see McKinnon [14] for more details on situations in which the Nelder–Mead algorithm fails). Enderlein et al. [15] used an MLE approach to distinguish between distinct states or molecules. [16] used MLE and iterative convolutions to fit the arrival time histograms to single exponential decay. Enderlein and Sauer [17] presented a pattern-matching procedure for identifying single molecules from a mixture of molecules, although the algorithm presented works best only if the lifetimes are already known. This is not applicable to the cases where we cannot experimentally separate the two distinct states of a complex. complex always exhibits mixed states because we cannot predetermine the lifetimes of the respective states. Edel et al. [11] developed a modified MLE method to compensate localized background fluorescence and instrument response function (IRF). However, this method focuses on fitting only the monoexponential decay curve.

Moreover, there are some non-MLE based parameter estimation methods in the literature. For example, Digman et al. [18] developed a phasor plot method and required labor-intensive visual inspection. Kim et al. [19] developed a promptness ratio method for estimating the lifetime.

This paper focuses on two issues, numerical stability and overfitting small data sets. Overfitting the data in this context can be described as yielding a model which gives very high probability to data similar to the observed data yet describing the true underlying generative process poorly. When fitting a mixture of exponential decay curves with binned data, the numerical optimization algorithm for finding MLE may not converge. Even if it converges, in practice the numerical optimization algorithm may converge to a value that is physically unreasonable. In addition, we show that the MLE’s for the mixture of exponential distributions can often overfit the data, hence giving estimates that appear satisfactory but fail to accurately represent the true parameter values.

To address these issues, we propose a novel method of estimating the parameters of the biexponential distribution using binned count data. The object is to find a generalization of the mono-exponential distribution whose pdf is flexible enough to well approximate the shape of a biexponential density curve. With this motivation in mind, we propose a new estimation method which utilizes the gamma distribution family, a family which contains that of monoexponential distributions. We show that our approach can successfully recover the parameters of the underlying biexponential distribution, while avoiding the inherent numerical instabilities involved with a mixture distribution. Our proposed estimation algorithm is robust, and is not likely to overfit the data.

The rest of the paper is organized as follows. We first present the model and the estimation method. We then demonstrate the performance of the proposed method through simulations in which data sets are generated using biexponential pdfs with varying parameters. We finally present results for real data analysis from the fluorophore Cy3, collected via single photon counting technique.

### Methods

Given the total number of photons, denoted by $n$, the photon counts in $m$ time intervals $\mathbf Y =\left\{ Y_1 \ldots Y_m \right\}$ has multinomial distribution $\mathbf Y \sim \text{ Multinom }(n, p_1, p_2, \ldots ,p_m)$ [2]. Let the delay time for the $i$-th photon be $X_i$. We assume that $X_i$’s are independently and identically distributed with some pdf $f_X(x)$ and cdf $F_X(x)=\int _{0}^x f_X(s)ds$. Then $p_j$ is the probability that $X_i$ falls in $\left( \delta (j-1), \delta j \right)$, where $\delta$ is the width of time interval (bin width). To ensure the constraint that $\sum _{j=1}^m p_j=1,$ we have the following normalized bin probabilities:

1 $$\begin{aligned} p_j &= \, Prob\left( \delta (j-1) \le X_i < \delta j \right) /Prob\left( 0 \le X_i < \delta m \right) \nonumber \\ &= \frac{F_X( \delta j)- F_X(\delta (j-1))}{F_X(\delta m)} \end{aligned}$$

As discussed before, we assume henceforth that $f_X$ is a biexponential pdf, and further from carefully controlled experimental conditions the main lifetime component is known. When fitting the model to the data via finding the MLEs of the parameters, instead of directly fitting a biexponential distribution, we propose to fit a gamma distribution to the $X_i$’s. In particular, the pdf of the biexponential distribution and of the gamma distribution are given respectively as

2 $$\begin{aligned} f_X(x)=\frac{c}{\tau _1}e^{-\frac{x}{\tau _1}}+\frac{1-c}{\tau _2}e^{-\frac{x}{\tau _2}} \end{aligned}$$

3 $$\begin{aligned} g_X(x)=\frac{1}{\left( \frac{\tau _{\gamma }}{\alpha }\right) ^\alpha \Gamma (\alpha )}x^{\alpha -1}e^{-\frac{\alpha x}{\tau _{\gamma }}}. \end{aligned}$$

The gamma distribution is chosen because it avoids the numerical instability found in practice when finding MLEs of the parameters in a mixture distribution and is flexible enough to approximate the biexponential distribution while being less likely than biexponential to overfit small data sets. It is worth noting that when $\alpha =1$ and either $c\in \{0,1\}$ or $\tau _1=\tau _2$, the gamma distribution is exactly the biexponential distribution (and both equal the monoexponential). Also of note is that although $g_X(x)$ diverges as $x\rightarrow 0$ (for $0<\alpha \le 1$, which we restrict to be the case), this is in practice negligible since the probability of $x$ being in a neighborhood around zero goes to zero as the neighborhood itself shrinks to zero, i.e., $\mathbb {P}(x<\epsilon )\rightarrow 0$ as $\epsilon \rightarrow 0$.

We estimate the parameters $(\alpha ,\tau _{\gamma })$ of the hierarchical model via maximum likelihood method through the minimization of

4 $$\begin{aligned} -loglik(\varvec{\alpha ,\tau _{\gamma }};\varvec{y}) = n\log (G_X(\delta m)) - \sum _{j=1}^m y_j \log (G_X(\delta j)-G_X(\delta (j-1))) + C \end{aligned}$$

where $G_X$ is the cumulative distribution function corresponding to the gamma distribution in (3), $y_j$ is the observed photon count in the $j$th time interval, and $C$ is a constant; $G_X$ implicitly depends on the parameters $(\alpha ,\tau _{\gamma })$. The minimization of (4) is carried out using the Nelder–Mead algorithm.

The estimates of parameters in the gamma distribution are then converted to those in the biexponential distribution with equal mean and variance, i.e., we match the first and second moments of the biexponential and the gamma pdf’s. Since there are two remaining unknown parameters in the biexponential distribution, we solve the system of equations satisfying

5 $$\begin{aligned} \mathbb {E}(X^k | X \sim \text{ Gamma }) = \mathbb {E}(X^k | X \sim \text{ Biexp }) \quad \text{ for } k=1,2. \end{aligned}$$

By solving this system of equations, we are matching the expected value of $X^k$ for $k=1,2$. This is equivalent to the system of equations given by the derivatives of the moment generating functions as following

6 $$\begin{aligned} M^{(k)}_{X,\gamma }(0)=M^{(k)}_{X,BE}(0) \quad \text{ for } k=1,2. \end{aligned}$$

where

7 $$M_{X,\gamma }(t) \, = \, \left(1-\frac{\tau _{\gamma }}{\alpha }t\right)^{-\alpha },$$

8 $$M_{X,BE}(t) \, = \, c(1-\tau _{1}t)^{-1}+(1-c)(1-\tau _{2}t)^{-1},$$

and where $M_{X,\cdot }^{(k)}$ is the* k*th derivative of $M_{X,\cdot }$ with respect to $t$. The closed form solutions using the MLEs from fitting the gamma distribution to approximate the parameters of the biexponential distribution are

9 $$\begin{aligned} \hat{\tau }_2 &= \, \frac{2\alpha \tau _{\gamma }\tau _1-(1+\alpha )\tau _{\gamma }^2}{2\alpha \tau _1-2\alpha \tau _{\gamma }}, \nonumber \\ \hat{c} &= \, \frac{(1-\alpha )\tau _{\gamma }^2}{2\alpha \tau _1^2-4\alpha \tau _{\gamma }\tau _1+(1+\alpha )\tau _{\gamma }^2}. \end{aligned}$$

Note that, while the focus of this paper is the context where one lifetime component is known, this new method of estimating the parameters of the biexponential distribution can be easily extended to the cases where the main lifetime component is unknown. We can accomplish this by simply matching the first three moments, i.e. letting $k=1,2,3$ in Eq. (5). If this is the case, the conversion equations become

10 $$\begin{aligned} \widehat{\tau }_1 &= \, \frac{\tau _{\gamma }\left( \sqrt{2(2-\alpha )(\alpha +1)}-\alpha -1 \right) }{\sqrt{2(2-\alpha )(\alpha +1)}-4\alpha +2w} \nonumber \\ \widehat{\tau }_2 &= \, \frac{\tau _{\gamma }\left( \sqrt{2(2+\alpha -\alpha ^2)}+2(\alpha +1) \right) }{6\alpha }\nonumber \\ \widehat{c} &= \, \frac{\left( \frac{\alpha +1}{2\alpha }\right) \tau _{\gamma }^2-\tau _2^2}{\tau _1^2-\tau _2^2}. \end{aligned}$$

To the authors’ knowledge, single molecule lifetime analyses involve only as complex a model as a biexponential decay. However, there is no theoretical reason as to why our method could not be applied to an exponential mixture decay curve with greater than two components, though as the number of components in the exponential mixture model increases, the algebra in finding the closed form solution quickly becomes tedious and impractical. In general, assuming no lifetime components known *a priori*, the system of equations needed to be solved for a $M$ component exponential mixture model is $M^{(k)}_{X,\gamma }(0)=M^{(k)}_{X,BE}(0)$ for $k=1,2,\ldots ,2M-1$.

### Results

The performance of the proposed method can be assessed in two ways. First, we compare the estimates of the second unknown lifetime component $\tau _2$ to the true value. Second, we evaluate the overfitness of the estimation by comparing how well the estimates fit the data to how close the estimates are to the truth. To this end we use two quantitative measures, Pearson’s $\chi ^2$ statistic and the Hellinger Distance (see., e.g., [20]). We compute the Pearson’s $\chi ^2$ statistic based on the multinomial distribution of the binned count data to determine how closely our fitted model fits the data. It is computed as

11 $$\begin{aligned} \chi ^2 = \sum _{j=1}^m \frac{(y_j - \mathbb {E}(y_j))^2}{\mathbb {E}(y_j)} \end{aligned}$$

where $$\mathbb {E}(y_j)$$ is

12 $$\begin{aligned} \mathbb {E}[Y_j]=n \cdot p_j = n \cdot \frac{F_X \left( \delta j \right) - F_X \left( \delta (j-1) \right) }{F_X\left( \delta m \right) }. \end{aligned}$$

To measure how close our estimated biexponential curve is to the true curve we use Hellinger’s Distance. This is a metric commonly used in the statistical literature to compare two different pdf’s. By using Hellinger’s Distance to compare the true curve and the estimated curve, we see how close to the truth our estimations are. Hellinger Distance can by computed as

13 $$\begin{aligned} H(f,h)\propto & {} \left\{ \int \left( f^{\frac{1}{2}} - h^{\frac{1}{2}} \right) ^2 \right\} ^{\frac{1}{2}} \nonumber \\\propto & {} \sqrt{ 2-2\int (fh)^{\frac{1}{2}} } \end{aligned}$$

where $f$ represents a curve fit from the small data sets, and $h$ represents the true (or our best approximation to the true) data generating process. We do not have a closed form for the Hellinger Distance between two biexponential distributions; however, since

$$\begin{aligned} \int (fh)^{\frac{1}{2}}= \int \sqrt{\frac{f}{h}}h = E_h \left( \sqrt{\frac{f}{h}} \right) \end{aligned}$$

and since one can make random draws from $h$, it is straightforward to use the Monte Carlo estimate of the Hellinger Distance $H(f,h)$ for any $f$. That is, for sufficiently large N,

14 $$\begin{aligned} H(f,h) \approx \sqrt{2- \frac{2}{N} \sum _{i=1}^N \left( \sqrt{\frac{f(z_i)}{h(z_i)} }\right) } \end{aligned}$$

where $z_1,z_2, \ldots , z_N$ are independent draws from $h$.

### Simulation results

Biexponential data were simulated as follows. In the biexponential distribution, the first lifetime component $\tau _1$ was fixed at 1,500, and assumed known when fitting the biexponential distribution directly by maximizing the likelihood and when fitting the biexponential distribution indirectly by using the gamma conversion method; the first component weight $c$ took values in $\{ 0.60, \, 0.75, \, 0.90\}$; the second lifetime component $\tau _2$ took values equaling $k\tau _1$, for $k$ in {0.500, 0.800, 0.900, 0.950, 0.990, 1.01, 1.05, 1.10, 1.20, 2.00}; the bin width $\delta$ was set to be 50. We generated 1,000 data sets of 50 photons for each of the 30 configurations. For each data set we estimated the lifetime parameter values by fitting the biexponential directly and also by using our proposed approach. In both cases optimization was performed by using the Nelder–Mead algorithm, setting the maximum number of iterations to be 10,000 and the relative convergence tolerance to be $1\times 10^{-8}$. In the former case, we attempted to fit the data using 25 different starting values of $c$ and initializing $\tau _2$ to be equal to the mean of the bin counts (i.e., $\sum _j (jy_j)/\sum _{\ell }y_{\ell }$). In the latter case we initialized $\alpha =0.5$, and $\tau _{\gamma }$ was initialized similarly to $\tau _2$ when fitting the biexponential directly. We note here that to find good solutions from the optimization algorithm it was necessary to use multiple starting points for fitting the biexponential directly whereas this was not necessary with our method; in particular, without using multiple initialization points for fitting the biexponential distribution directly we would often fail to converge or obtain poor estimates. Out of the 30,000 simulated data sets, attempting to fit the biexponential model directly failed to converge in 14,272 instances even while using multiple starting points, as opposed to 2,160 instances when using the proposed gamma method using only one starting point.

**Figure 1:**
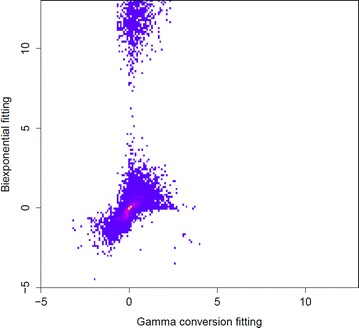
Parameter estimates from simulated data. Two-dimensional histogram of $\log (\widehat{\tau }_2/\tau _2)$, where $\widehat{\tau }_2$ us the estimate from either using the biexponential pdf directly (*vertical axis*) or the gamma conversion method (*horizontal axis*); the estimates are aggregated over 30 varying true values of $c$ and $\tau _2$. Intensity is graded from* blue* (lowest) to* yellow* (highest), white indicating no counts.

To compare the performance of the methods, we focus on the data sets in which both methods converged and use the quantity $\log (\widehat{\tau }_2/\tau _2)$ (base $e$) as a benchmark. Figure 1 shows the 2-dimensional histogram of these estimates, obtained both from directly fitting the biexponential distribution (vertical axis) and from using the gamma conversion method (horizontal axis). It can be seen from this plot that when fitting the biexponential directly, even among the data sets in which convergence was reached, there are a large number of occasions where the estimated values of $\tau _2$ are physically unreasonable, yet the gamma conversion method provides reasonable answers. This can be further seen by looking at, e.g., the 5th and 95th quantiles of $\widehat{\tau }_2/\tau _2$, which were 0.23 and 202,000 respectively when fitting the biexponential directly and 0.35 and 5.2 respectively when applying the gamma conversion method. These numbers suggest the estimates obtained from directly fitting biexponential distribution are numerically instable compared to those obtained from gamma conversion method.

In reality, many of these results yielding extremely large estimates simply would not be accepted in practice. Instead, an artificial ceiling may be put on the lifetime estimates. When we do this in our simulation study, using a cap of 100 ns, our results lead to the same conclusions. To give a brief summary of these slightly modified results, we computed the mean square error (MSE), which is the average of $(\widehat{\tau }_2-\tau _2)^2$, for the direct fitting of the biexponential ($1{,}500\,ns^2$) and for our proposed gamma conversion method ($55\,ns^2$); clearly even with this truncation of extremely high estimates, our proposed method is performing much better.

**Figure 2:**
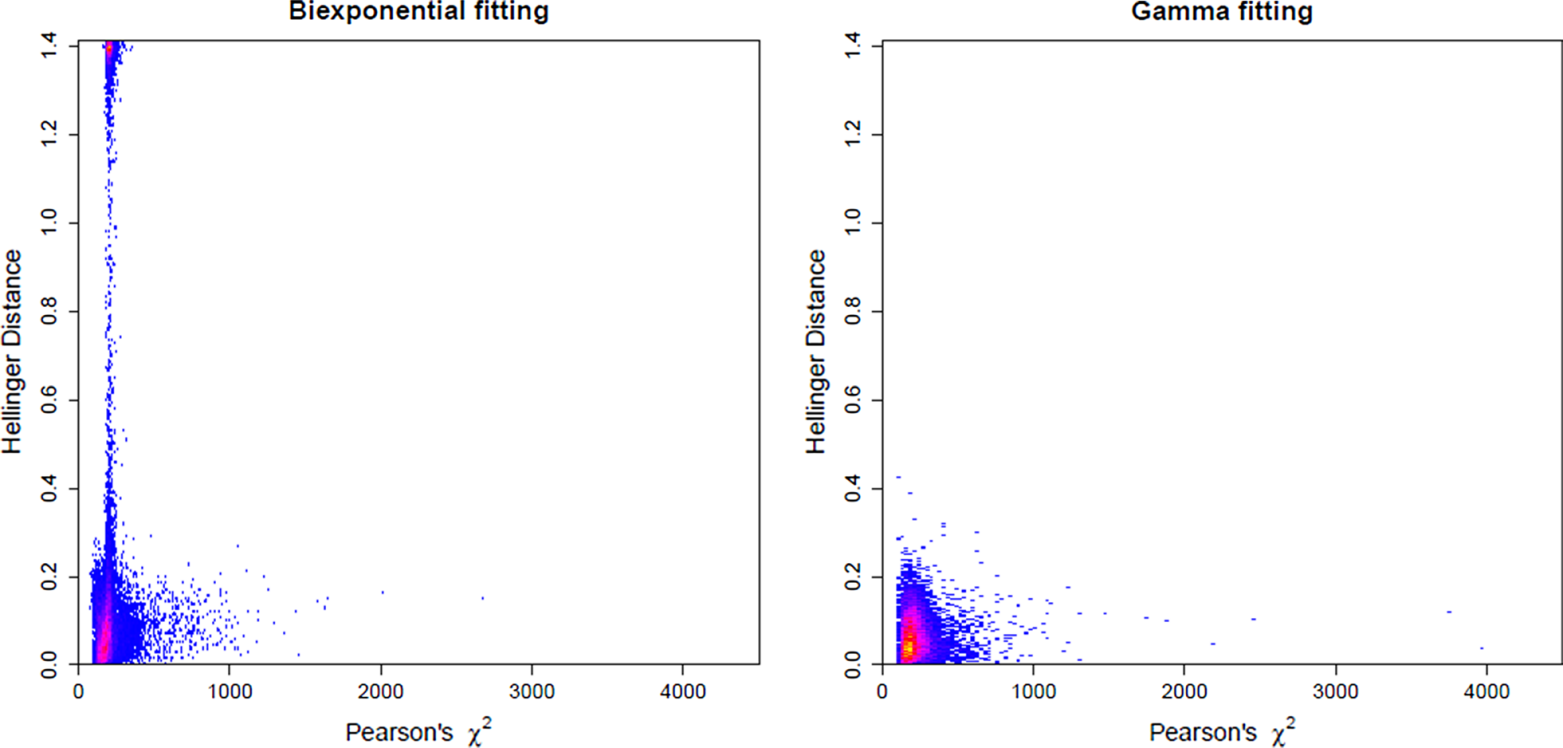
Evidence of overfitting from simulated data. Two-dimensional histogram for Hellinger Distance (*vertical axis*) and Pearson’s $\chi ^2$ statistic (*horizontal axis*) for simulated data, where the plotted values have been aggregated over varying true values of $c$ and $\tau _2$. Fitting the biexponential directly gives the plot on the* left*, and using gamma conversion method gives the plot on the* right*. Intensity is graded from* blue* (lowest) to* yellow* (highest), white indicating no counts.

To evaluate the overfitting problem, we compute the Hellinger Distance and Pearson’s $\chi ^2$ statistics. For each of the simulated data sets in which both methods converged, these two values were computed by fitting the biexponential distribution directly and also by using the gamma conversion method. Figure 2 gives the two-dimensional histogram of these values, where the plot on the left corresponds to fitting the biexponential directly and the plot on the right corresponds to using the gamma conversion method. We see that fitting the biexponential directly, in a large number of the data sets, yields estimates which fit the data quite well, as evidenced by a small $\chi ^2$ value, but are far from the truth, as evidenced by a large Hellinger Distance. Using the gamma conversion method eliminates this overfitting problem, as evidenced by the observations that all the Hellinger Distance values are small.

Figures 1 and 2 broken down by simulation configuration are given in the Additional files 1, 2, 3 and 4. What is evident is that while the problems of numerical instability and overfitting which arise from fitting the biexponential distribution directly are milder in some configurations than others, these problems do in fact exist for each configuration, while our proposed approach greatly ameliorates these issues. When $\tau _1$ was treated as unknown we obtained similar results. See Additional files 1, 2, 3 and 4 for these results.

### Fluorophore Cy3 results

**Figure 3:**
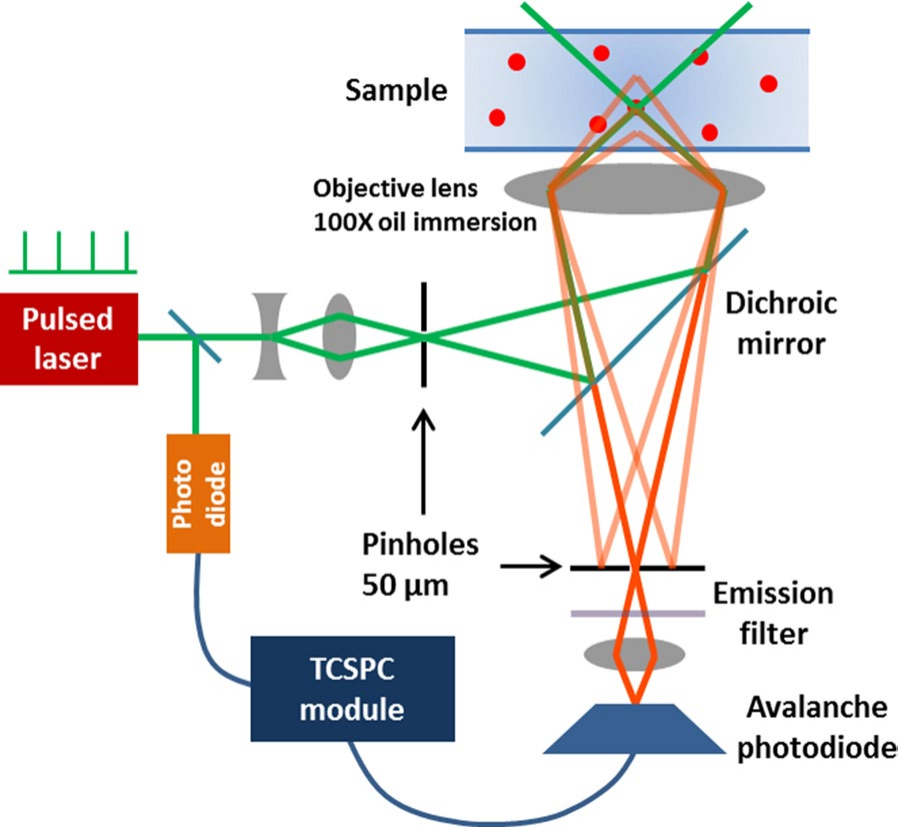
Experimental schematic. Schematic of the confocal-TCSPC setup.

Single molecule fluorescence lifetime was measured as follows. We used a confocal microscope setup to minimize the detection volume. A DNA strand labeled with Cy3

(5′-Cy3-TATTATATAAGTAATAAATA-3′) was excited by 532 nm quasi-continuous pulsed laser (Vanguard VGND2000-76-HM532, Spectra-Physics), which has 12 ps pulse width. These make a broadening of about 5% of the lifetime we are measuring. While there exist methods to deconvolute the IRF and our software is capable of doing it, we did not do it for this study as the broadening is negligible compared to the broadening that we are dealing with, coming from the small number of photons. Further, we considered only the time window after the peak of the lifetime decay in order that our fitting would not be seriously affected by the IRF broadening. The emitted photons passed through the objective lens, dichroic mirror, emission filter, and focusing lens, and then were collected at the avalanche photodiode (PD5CTC, Micro Photon Devices). Out-of-focus signal was rejected by use of the pinhole pair in this confocal setup (see Figure 3).

The excitation pulses were branched to a photodiode for synchronization. Time delay between the signals from the avalanche and sync photodiodes was measured by the time correlated single photon counter (SPC630, Becker&Hickl GmbH). Figure 3 shows a schematic of that described above. We used 2 nM of fluorophores for detecting fluorescence from diffusing molecules. We set it up such that it gives the APD counting rate smaller than $10^5$/s. Considering that the excitation pulse repeats at 80 MHz (i.e. 12.5 ns), this corresponds to detecting less than one photon every 800 pulses on average. The probability of detecting more than two photons (from two different molecules) from a single pulse is less than 1/800. As we used only 50 photons per histogram and also the pulse interval of 12.5 ns much longer than the decay time, there will be practically no photon that is not coming from the latest excitation pulse. Thus we confirm that we are measuring tightly correlated photon emission from excited single molecules. The data from SPC630 were collected until desired number of photons were detected and then plotted as a lifetime histogram with appropriate bin sizes. See [1] for more details.

**Figure 4:**
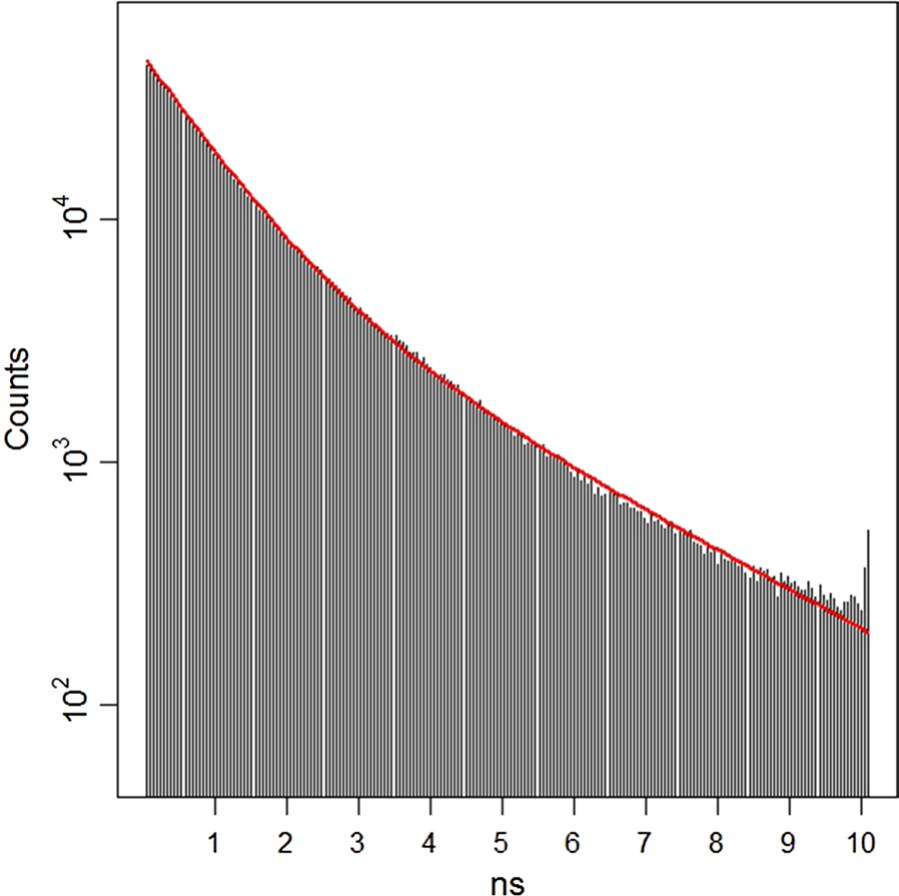
Cy3 data. Histogram of Cy3 fluorophore data on log scale, with estimated biexponential distribution superimposed.

One large data set ($\approx$1.2 million photons) was obtained from the above experiment. The bin width used was 12.5 ns/256 = 48.8 ps; the maximum amount of time in the observation window is 10,101.6 ps (207 bins). The biexponential distribution was fit directly to obtain $\tau _1=927.4$ ps, $\tau _2=2739$ ps, and $c=0.6171$. These components are comparable to those found by [21], who obtained the estimates $\tau _1=530$ ps, $\tau _2=2000$ ps, and $c=0.51$. The main component here was $\tau _1$, and for the remainder of the analysis we assumed this value known, while $c$ and $\tau _2$ remained to be estimated, treating the estimates of 2,739 ps and 0.6171 as the “true” values for the rest of the analysis. Figure 4 gives the histogram of the large data set, with the estimated biexponential curve superimposed. Next we sampled without replacement from the large data set to obtain 2,586 small data sets of sample size 50. Photon decay curves were estimated from these small data sets using both the biexponential pdf directly and the gamma conversion method, and the results were compared to the true values. The intialization of the optimization algorithms was the same as that done for the simulated data.

**Figure 5:**
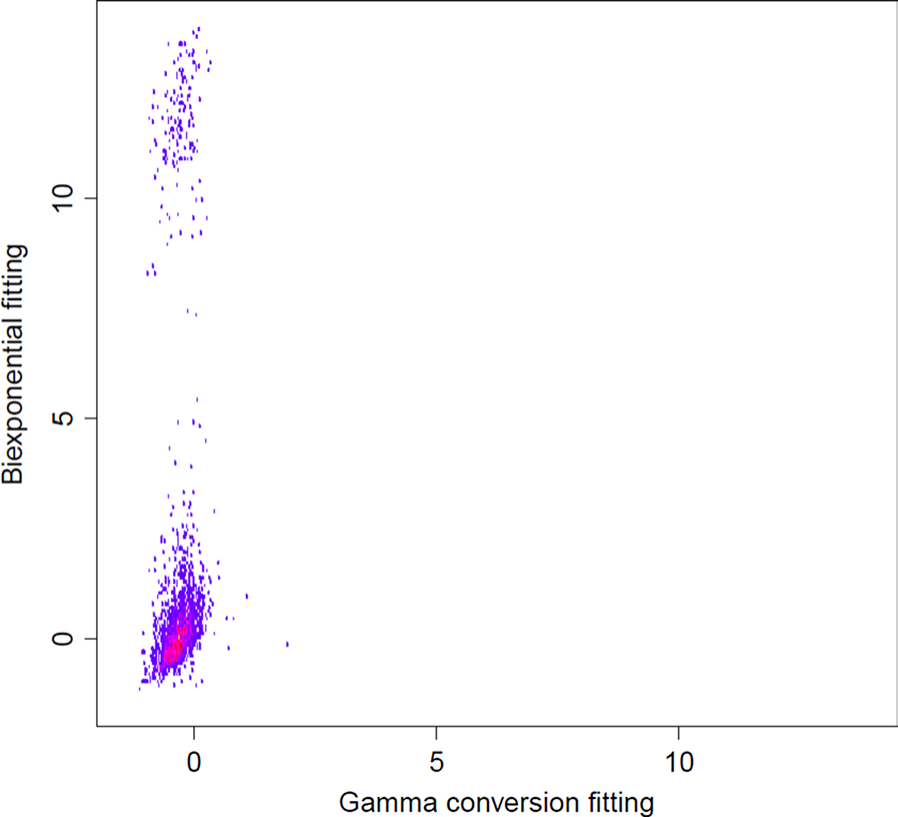
Parameter estimates from Cy3 data. Two-dimensional histogram of $\log (\widehat{\tau }_2/\tau _2)$, where $\widehat{\tau }_2$ us the estimate from either using the biexponential pdf directly (*vertical axis*) or the gamma conversion method (*horizontal axis*); the estimates are obtained by fitting the fluorophore Cy3 data. Intensity is graded from* blue* (lowest) to* yellow* (highest),* white* indicating no counts.

Attempting to fit the biexponential pdf directly led to 364 instances of failure to converge, as opposed to just one using the gamma conversion method. For each of the small data sets where both methods converged, the quantity $\log (\widehat{\tau }_2/\tau _2)$ was computed for $\widehat{\tau }_2$, which was estimated by fitting the biexponential directly and also by the gamma conversion method. Figure 5 is a two-dimensional histogram of these values, where the vertical axis corresponds to fitting the biexponential directly and the horizontal axis corresponds to the gamma conversion method. From this figure, it is clear that there are a large number of data sets in which the estimates of $\tau _2$, obtained directly by fitting the biexponential distribution, are physically unreasonable values, yet the gamma conversion method provides reasonable answers. The 5th and 95th quantiles of $\widehat{\tau }_2/\tau _2$ were 0.55 and 67,000 from fitting the biexponential directly and 0.49 and 1.1 when using the gamma conversion method. These numbers suggest that the gamma conversion method is giving more stable results than that obtained from fitting the biexponential distribution directly.

As with the simulation study, we again applied a ceiling of 100 ns to the extremely high estimates of $\tau _2$. Again the conclusions were the same for these modified results. The MSE for directly fitting the biexponential was $725\,ns^2$ and for the gamma conversion method was $0.89\,ns^2$.

**Figure 6:**
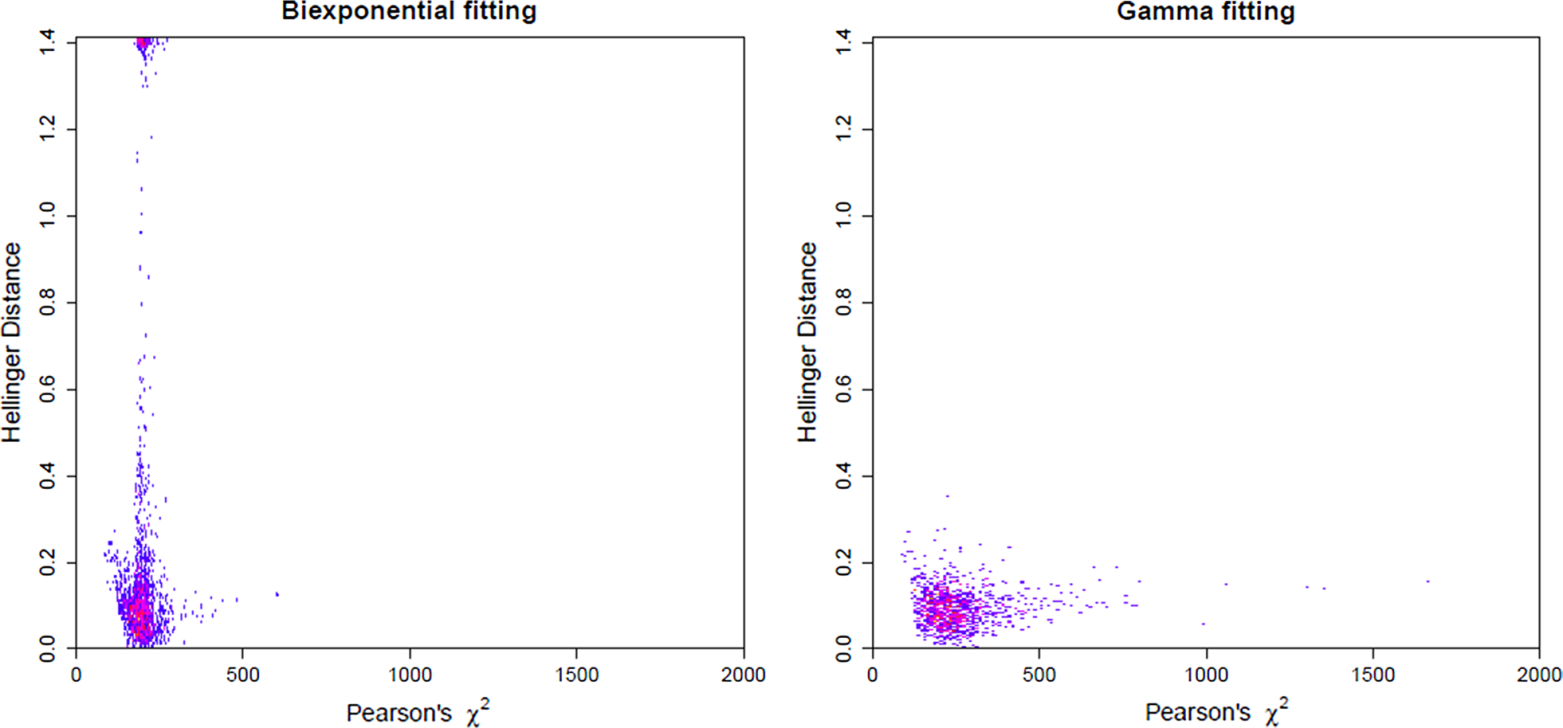
Evidence of overfitting the Cy3 data. Two-dimensional histogram for Hellinger Distance (*vertical axis*) and Pearson’s $\chi ^2$ statistic (*horizontal axis*) for fluorophore Cy3 data. Fitting the biexponential directly gives the plot on the* left*, and using gamma conversion method gives the plot on the* right*. Intensity is graded from* blue* (lowest) to* yellow* (highest),* white* indicating no counts.

We also computed, for each of the small data sets, the Hellinger Distance and $\chi ^2$ statistic for both methods. Figure 6 is the two-dimensional histogram of Hellinger Distances vs. $\chi ^2$ statistics, where the plot on the left corresponds to those values computed when fitting the model to the biexponential distribution directly and the plot on the right is when using the gamma conversion method. We observe, just as in the simulations, that when fitting the biexponential directly, in a large number of the smaller data sets, we have estimates that fit the data quite well, as evidenced by small $\chi ^2$ values, but the estimated decay curve is far from the truth, as evidenced by a large Hellinger Distance. Using the gamma conversion eliminates this overfitting problem, as evidenced by the fact that all the Hellinger Distance values are small.

Similar results were obtained for the fluorophore Cy3 data when $\tau _1$ was assumed to be unknown. See Additional files 1, 2, 3 and 4 for these results.

### Conclusion

In the single-molecule fluorescence lifetime experiments, it is of great interest to study the photon delay time. In particular, we are interested in fitting a mixture of exponential model to the photon count data. However, directly fitting a mixture of exponential model may lead to numerical optimization problems, whether that be failure to converge or convergence to local optima resulting in physically unreasonable values or overfitting. In this paper, we proposed the gamma conversion method, where we first fit a gamma distribution to the data and then, via moment matching, estimate biexponential parameters. In this manner both the numerical instability and the overfitting problems are avoided.

The proposed method was evaluated using Pearson’s $\chi ^2$ statistic and the Hellinger Distance. As an alternative to Pearson’s $\chi ^2$ statistic and the Hellinger Distance, we could have compared the MSE, just as we did when we applied the ceiling to the lifetime estimates. Calculating the ratio of MSEs obtained from fitting the biexponentials directly and from our proposed method yielded a value of 5.6e10 for the real data example, and similar ratios were consistently found in all 30 simulation configurations. These observations suggest that the estimates obtained from gamma conversion significantly outperform those obtained from directly fitting biexponenetials.

Although the method was designed to analyze photon counts in single-molecule fluorescence lifetime experiments, the method may be applied to other problems involving fitting mixture of exponential distributions. Most FLIM measurements, however, have rather large number of photons ($\sim1{,}000$) for each pixel, and thus do not suffer from the overfitting or numerical instability issues highlighted here when discussing single molecule fluorescence lifetime data.

## Additional files

**Additional file 1:** Numerical results 1
**Additional file 2:** Numerical results 2
**Additional file 3:** Numerical results 3
**Additional file 4:** Numerical results 4
