# Supplementary material for: A parameter estimation method for fluorescence lifetime data
Source: BMC Res Notes. 2015 Jun 9;8:230. doi: 10.1186/s13104-015-1176-y (PMC4467687; doi:10.1186/s13104-015-1176-y)
Supplement: Supplementary file 1 — Additional file 1: Numerical results 1 [file 13104_2015_1176_MOESM1_ESM.pdf]

Supplementary Material: A Parameter Estimation Method for Single Molecule Fluorescence Lifetime Data

Dan Sewell,[†] Hajin Kim,[‡] Taekjip Ha,[‡] and Ping Ma[*,†]

Department of Statistics, University of Illinois at Urbana-Champaign, and Department of Physics, University of Illinois at Urbana-Champaign

E-mail: pingma@illinois.edu

[*]To whom correspondence should be addressed
[†]Department of Statistics,University of Illinois at Urbana-Champaign
[‡]Department of Physics, University of Illinois at Urbana-Champaign

Simulation Results by Parameter Configuration

Figures 1 to 5 show, for each of 30 parameter configurations, a two-dimensional histogram of the quantity $\log(\widehat{\tau}_2/\tau_2)$, where τ_2 is the true parameter value and $\widehat{\tau}_2$ is the estimate of τ_2; the vertical axes correspond to the estimates obtained from fitting the biexponential distribution directly, and the horizontal axes correspond to the estimates obtained from the gamma conversion method.

Figures 6 to 15 show, for each of the 30 parameter configurations, a two-dimensional histogram of Hellinger Distances vs. Pearson's χ^2 statistics. For each configuration there is a plot corresponding to the estimated curves from fitting the biexponential distribution directly (left) and to the estimated curves from the gamma conversion method (right). As mentioned in the main text, the Hellinger Distance is a metric between two probability distributions. Here the estimated curve is compared with the true curve, and hence a small (large) distance implies that the estimate is very close to (very far from) the true curve. Pearson's χ^2 statistic indicates how well the estimates fit the data. Therefore if an estimate is overfitting the data it will have a low χ^2 value but high distance from the truth.

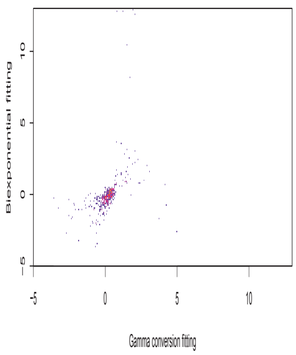

(a) $c = 0.6$, $k = 0.500$

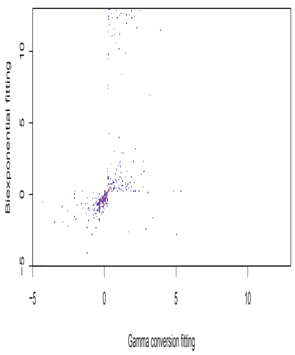

(b) $c = 0.6$, $k = 0.800$

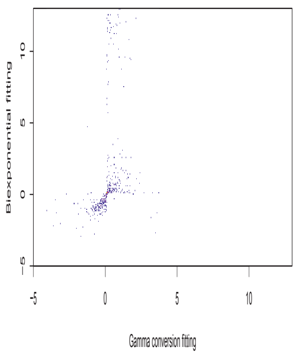

(c) $c = 0.6$, $k = 0.900$

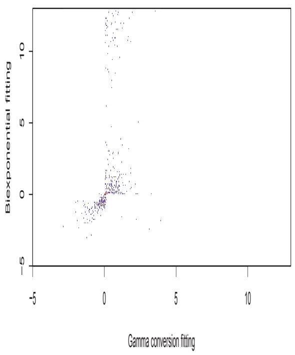

(d) $c = 0.6$, $k = 0.950$

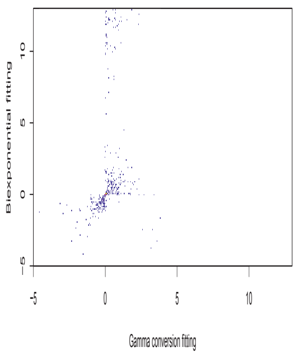

(e) $c = 0.6$, $k = 0.990$

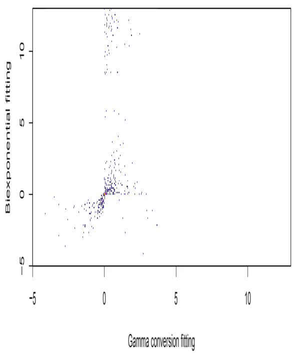

(f) $c = 0.6$, $k = 1.01$

Figure 1: Two-dimensional histograms of $\log(\widehat{\tau}_2/\tau_2)$, where $\widehat{\tau}_2$ is the estimate from either using the biexponential pdf directly (vertical axis) or the gamma conversion method (horizontal axis); each plot corresponds to varying true values of c and $\tau_2 = k\tau_1$. Intensity is graded from blue (lowest) to yellow (highest), white indicating no counts

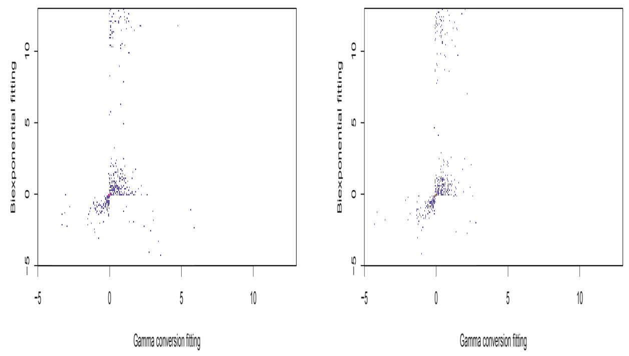

(a) $c = 0.6, k = 1.05$ (b) $c = 0.6, k = 1.10$

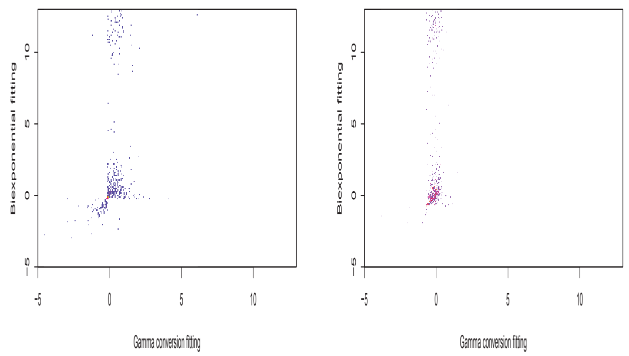

(c) $c = 0.6, k = 1.20$ (d) $c = 0.6, k = 2.00$

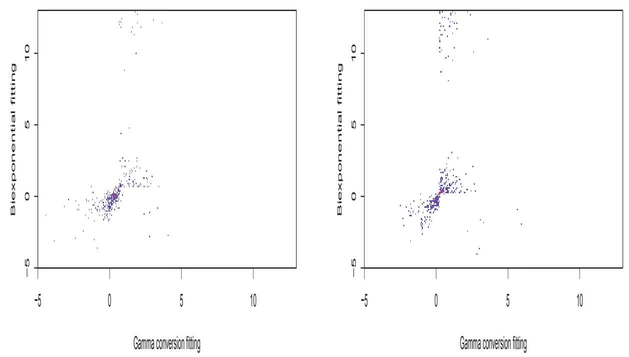

(e) $c = 0.75, k = 0.500$ (f) $c = 0.75, k = 0.800$

Figure 2: Two-dimensional histograms of $\log(\widehat{\tau}_2/\tau_2)$, where $\widehat{\tau}_2$ is the estimate from either using the biexponential pdf directly (vertical axis) or the gamma conversion method (horizontal axis); each plot corresponds to varying true values of c and $\tau_2 = k\tau_1$. Intensity is graded from blue (lowest) to yellow (highest), white indicating no counts

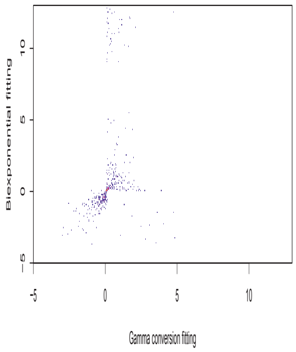

(a) $c = 0.75$, $k = 0.900$

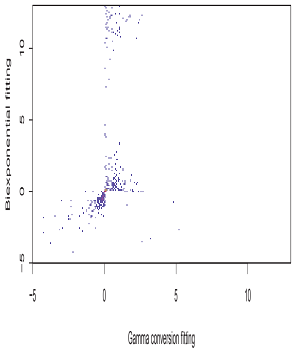

(b) $c = 0.75$, $k = 0.950$

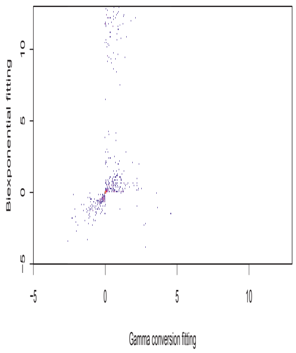

(c) $c = 0.75$, $k = 0.990$

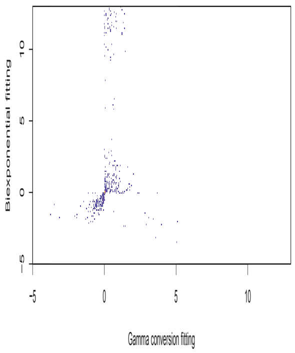

(d) $c = 0.75$, $k = 1.01$

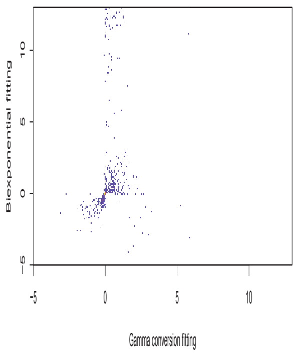

(e) $c = 0.75$, $k = 1.05$

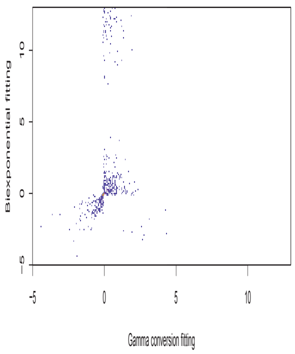

(f) $c = 0.75$, $k = 1.10$

Figure 3: Two-dimensional histograms of $\log(\widehat{\tau}_2/\tau_2)$, where $\widehat{\tau}_2$ is the estimate from either using the biexponential pdf directly (vertical axis) or the gamma conversion method (horizontal axis); each plot corresponds to varying true values of c and $\tau_2 = k\tau_1$. Intensity is graded from blue (lowest) to yellow (highest), white indicating no counts

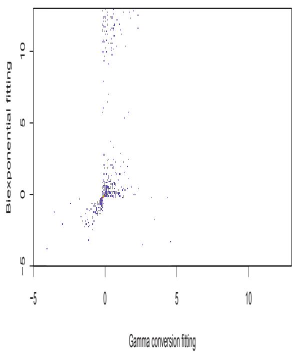

(a) $c = 0.75$, $k = 1.20$

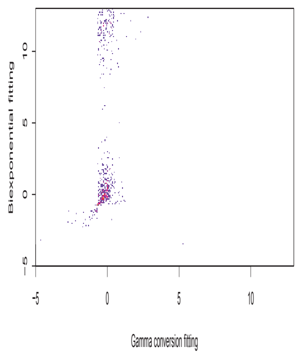

(b) $c = 0.75$, $k = 2.00$

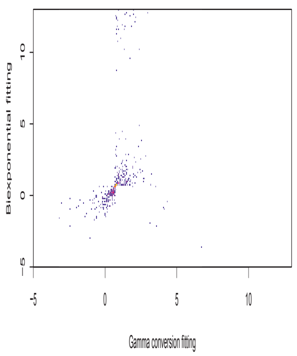

(c) $c = 0.90$, $k = 0.500$

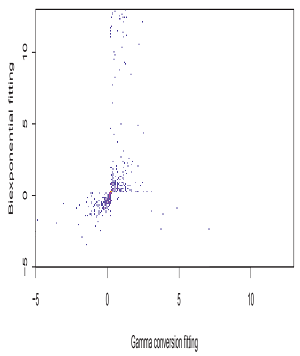

(d) $c = 0.90$, $k = 0.800$

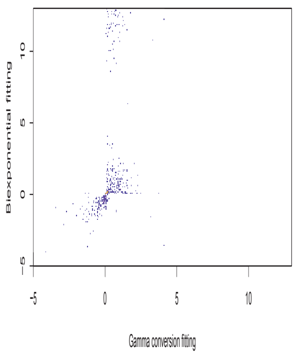

(e) $c = 0.90$, $k = 0.900$

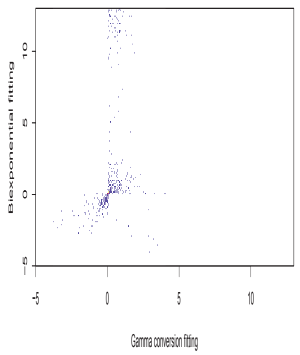

(f) $c = 0.90$, $k = 0.950$

Figure 4: Two-dimensional histograms of $\log(\widehat{\tau}_2/\tau_2)$, where $\widehat{\tau}_2$ is the estimate from either using the biexponential pdf directly (vertical axis) or the gamma conversion method (horizontal axis); each plot corresponds to varying true values of c and $\tau_2 = k\tau_1$. Intensity is graded from blue (lowest) to yellow (highest), white indicating no counts

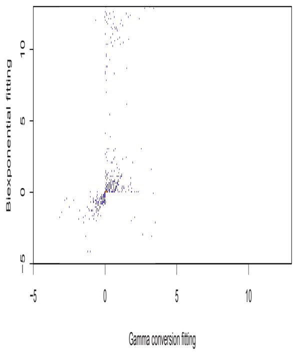

(a) $c = 0.90$, $k = 0.990$

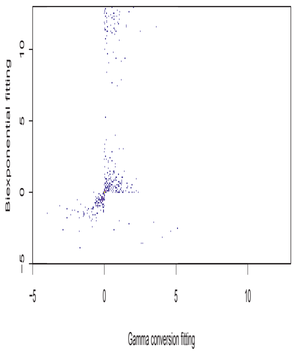

(b) $c = 0.90$, $k = 1.01$

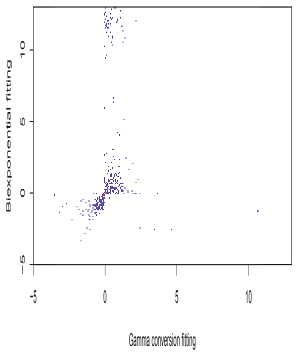

(c) $c = 0.90$, $k = 1.05$

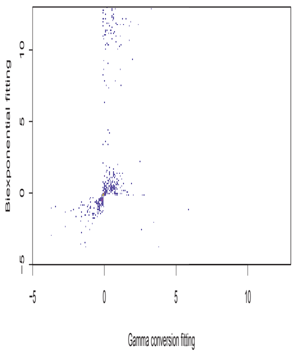

(d) $c = 0.90$, $k = 1.10$

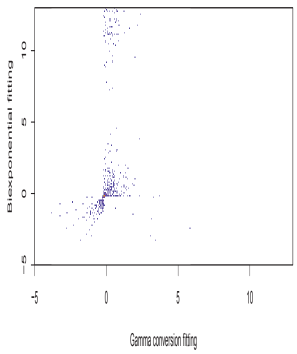

(e) $c = 0.90$, $k = 1.20$

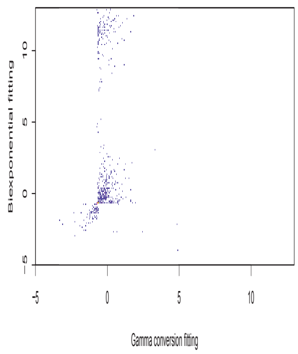

(f) $c = 0.90$, $k = 2.00$

Figure 5: Two-dimensional histograms of $\log(\widehat{\tau}_2/\tau_2)$, where $\widehat{\tau}_2$ is the estimate from either using the biexponential pdf directly (vertical axis) or the gamma conversion method (horizontal axis); each plot corresponds to varying true values of c and $\tau_2 = k\tau_1$. Intensity is graded from blue (lowest) to yellow (highest), white indicating no counts

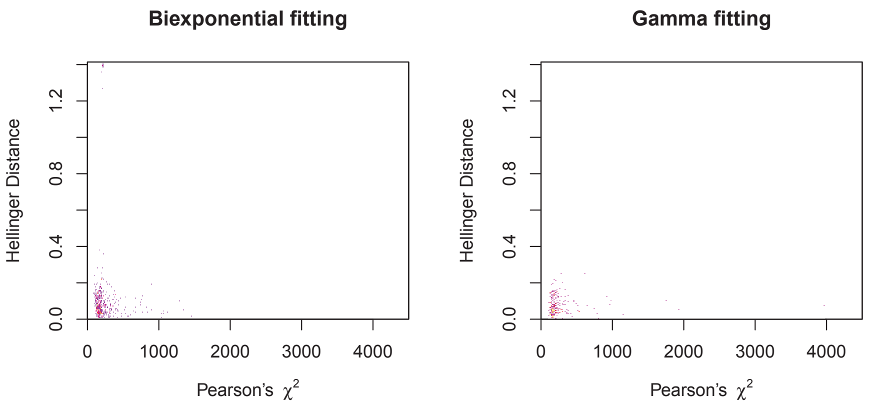

(a) $c = 0.6$, $k = 0.500$

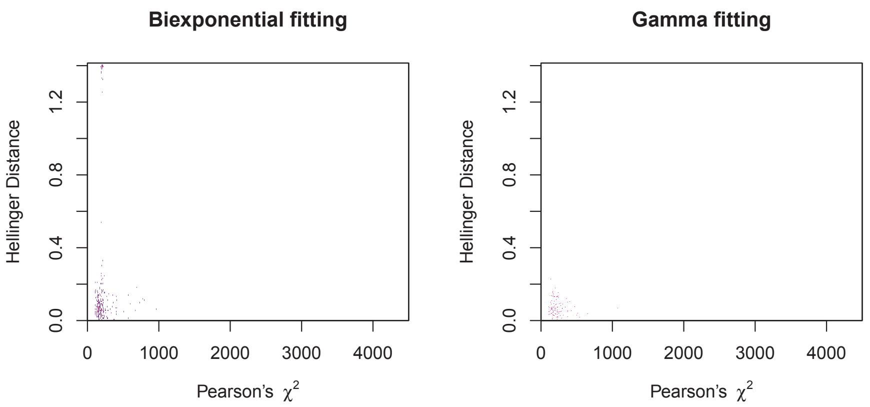

(b) $c = 0.6$, $k = 0.800$

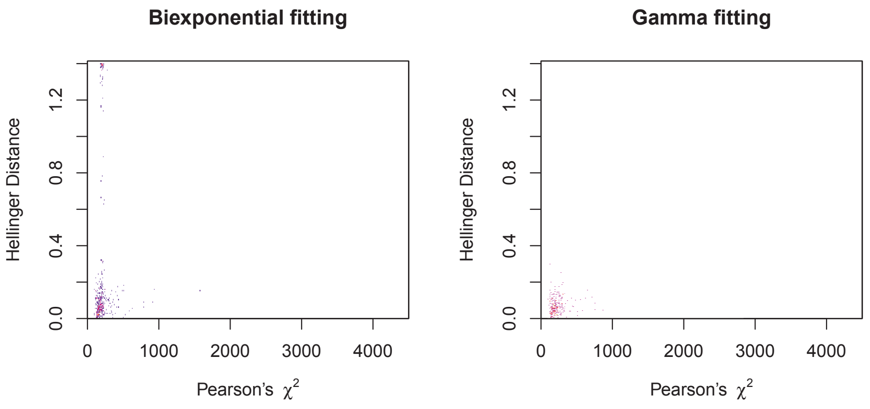

(c) $c = 0.6$, $k = 0.900$

Figure 6: Two-dimensional histogram for Hellinger Distance (vertical axis) and Pearson's χ^2 statistic (horizontal axis) for simulated data, where the plotted values have been aggregated over varying true values of c and τ_2. For each subfigure (a) through (c), fitting the biexponential directly gives the plot on the left, and using gamma conversion method gives the plot on the right. Intensity is graded from blue (lowest) to yellow (highest), white indicating no counts

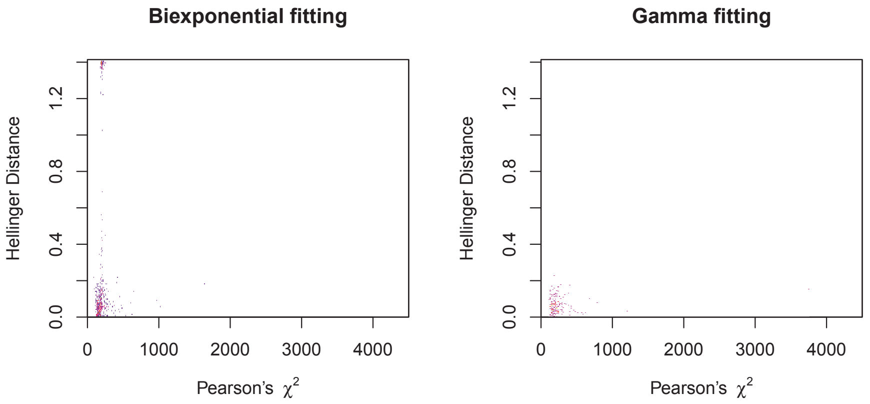

(a) $c = 0.6$, $k = 0.950$

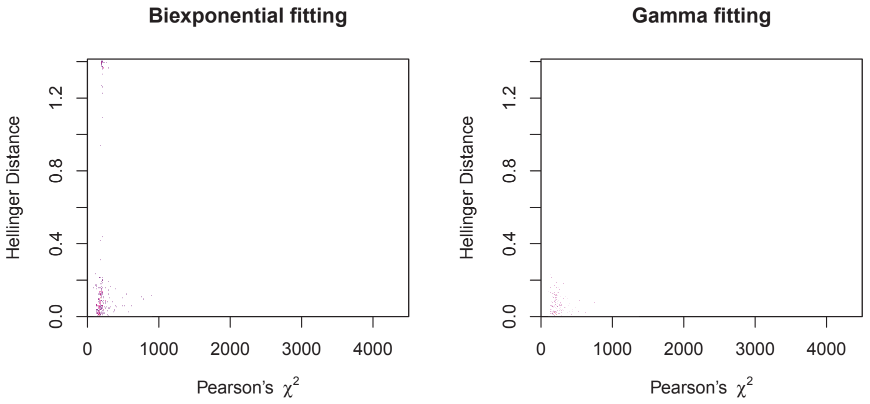

(b) $c = 0.6$, $k = 0.990$

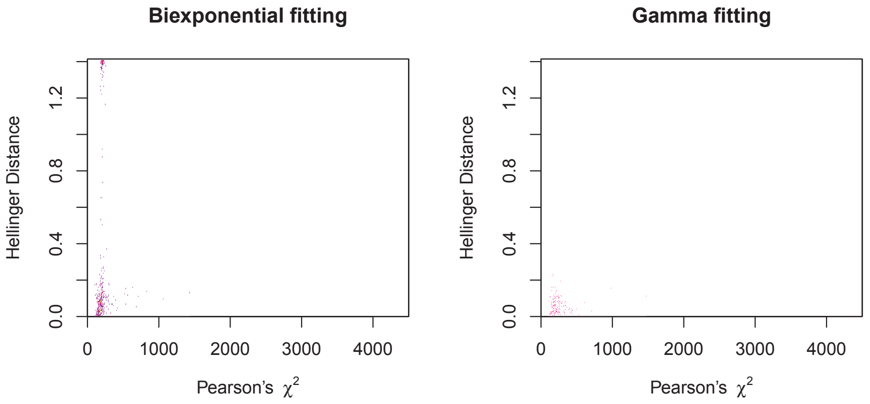

(c) $c = 0.6$, $k = 1.01$

Figure 7: Two-dimensional histogram for Hellinger Distance (vertical axis) and Pearson's χ^2 statistic (horizontal axis) for simulated data, where the plotted values have been aggregated over varying true values of c and τ_2. For each subfigure (a) through (c), fitting the biexponential directly gives the plot on the left, and using gamma conversion method gives the plot on the right. Intensity is graded from blue (lowest) to yellow (highest), white indicating no counts

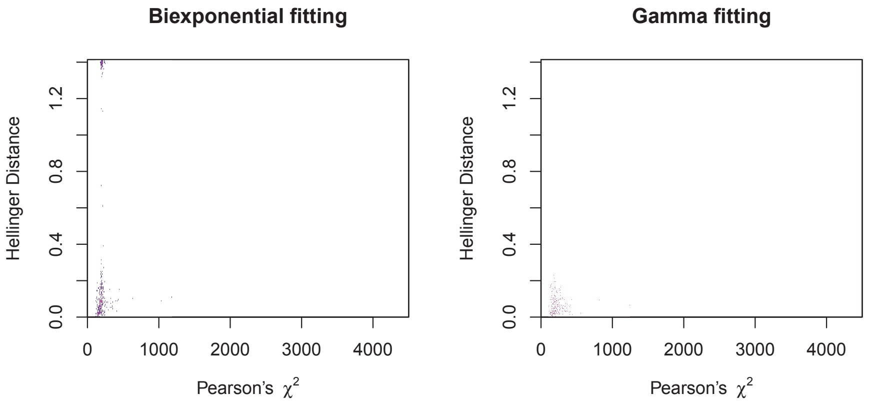

(a) $c = 0.6, k = 1.05$

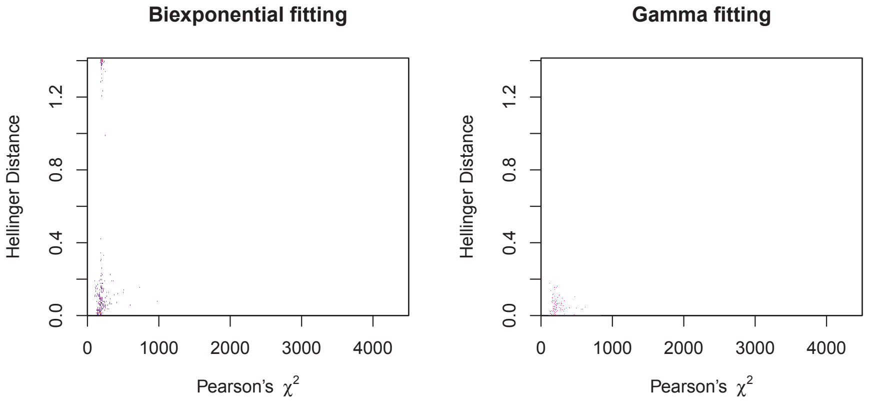

(b) $c = 0.6, k = 1.10$

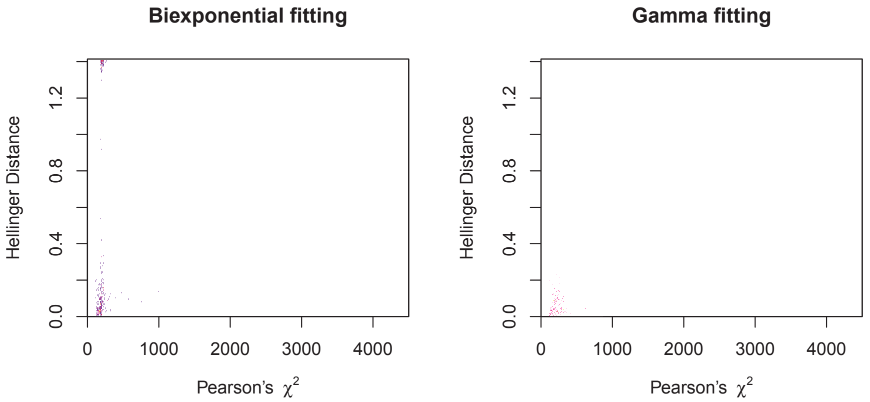

(c) $c = 0.6, k = 1.20$

Figure 8: Two-dimensional histogram for Hellinger Distance (vertical axis) and Pearson's χ^2 statistic (horizontal axis) for simulated data, where the plotted values have been aggregated over varying true values of c and τ_2. For each subfigure (a) through (c), fitting the biexponential directly gives the plot on the left, and using gamma conversion method gives the plot on the right. Intensity is graded from blue (lowest) to yellow (highest), white indicating no counts

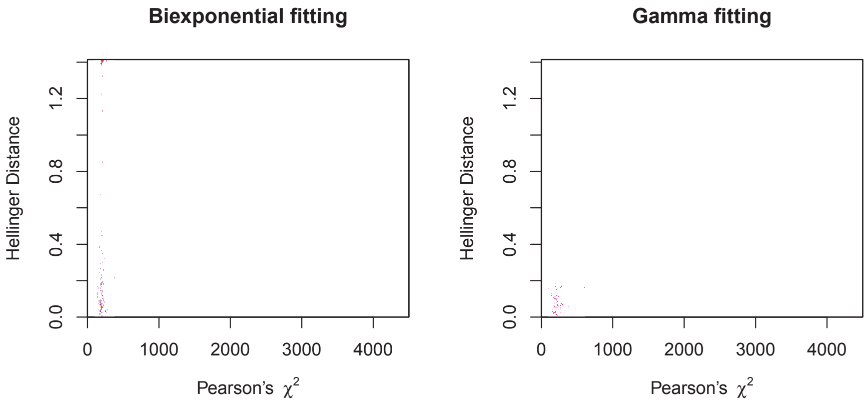

(a) $c = 0.6$, $k = 2.00$

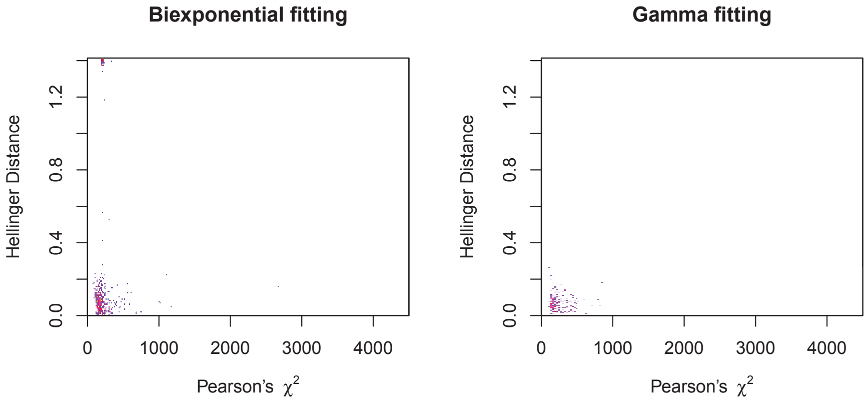

(b) $c = 0.75$, $k = 0.500$

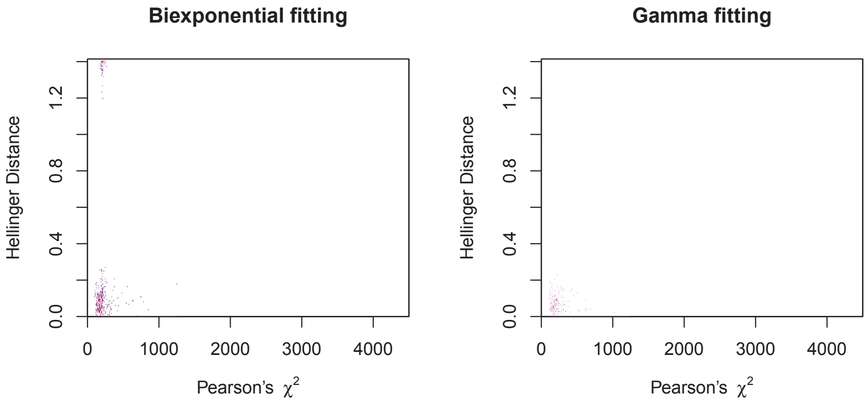

(c) $c = 0.75$, $k = 0.800$

Figure 9: Two-dimensional histogram for Hellinger Distance (vertical axis) and Pearson's χ^2 statistic (horizontal axis) for simulated data, where the plotted values have been aggregated over varying true values of c and τ_2. For each subfigure (a) through (c), fitting the biexponential directly gives the plot on the left, and using gamma conversion method gives the plot on the right. Intensity is graded from blue (lowest) to yellow (highest), white indicating no counts

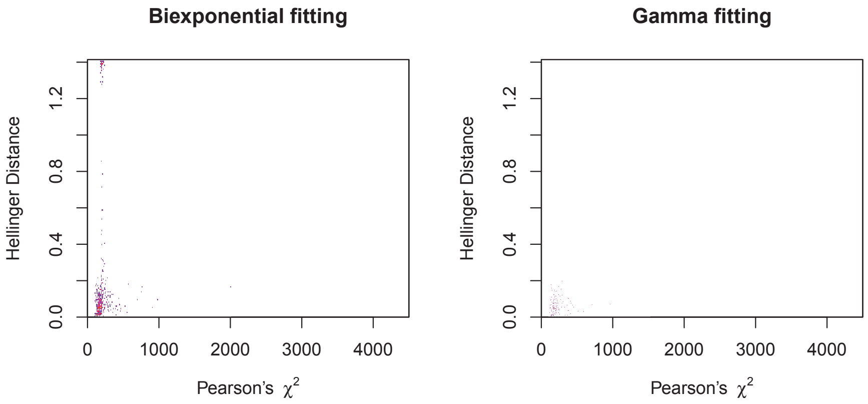

(a) $c = 0.75$, $k = 0.900$

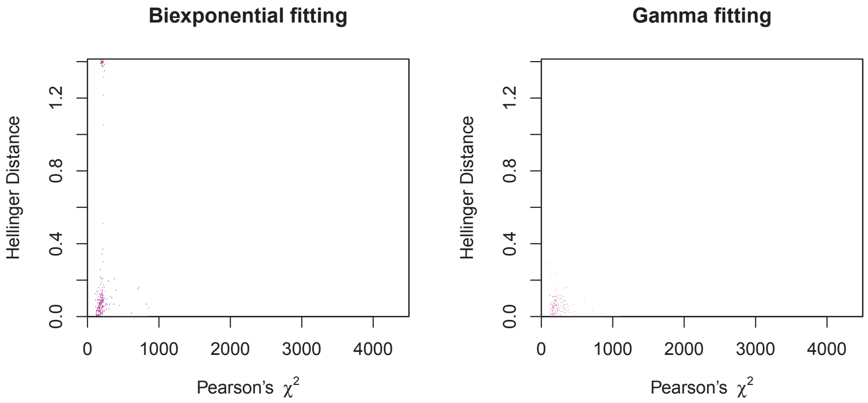

(b) $c = 0.75$, $k = 0.950$

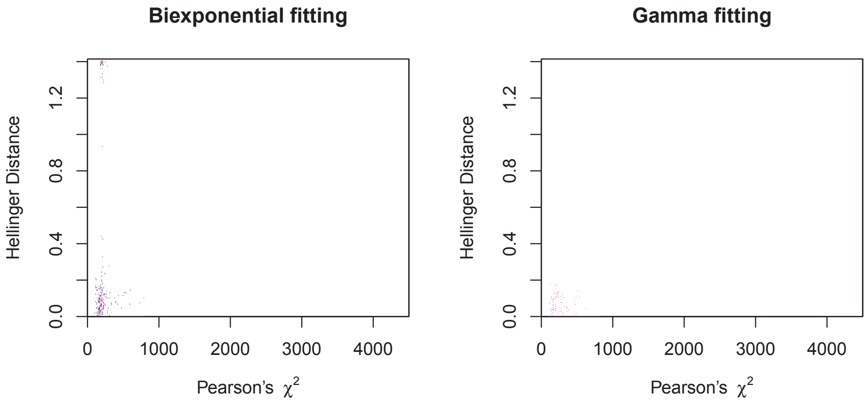

(c) $c = 0.75$, $k = 0.990$

Figure 10: Two-dimensional histogram for Hellinger Distance (vertical axis) and Pearson's χ^2 statistic (horizontal axis) for simulated data, where the plotted values have been aggregated over varying true values of c and τ_2. For each subfigure (a) through (c), fitting the biexponential directly gives the plot on the left, and using gamma conversion method gives the plot on the right. Intensity is graded from blue (lowest) to yellow (highest), white indicating no counts

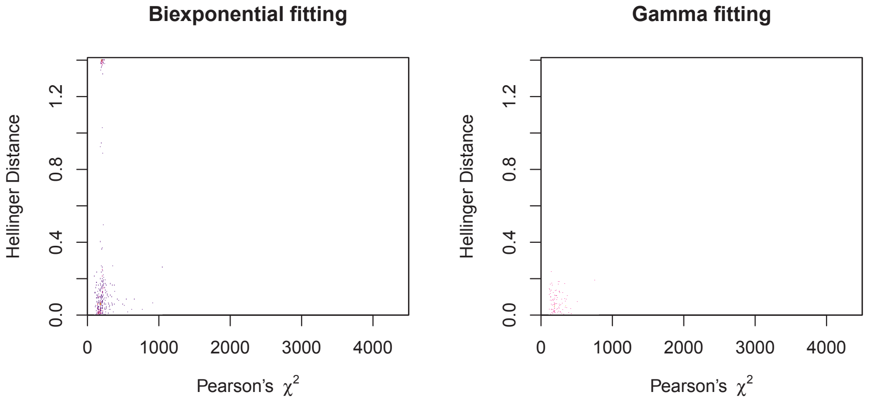

(a) $c = 0.75$, $k = 1.01$

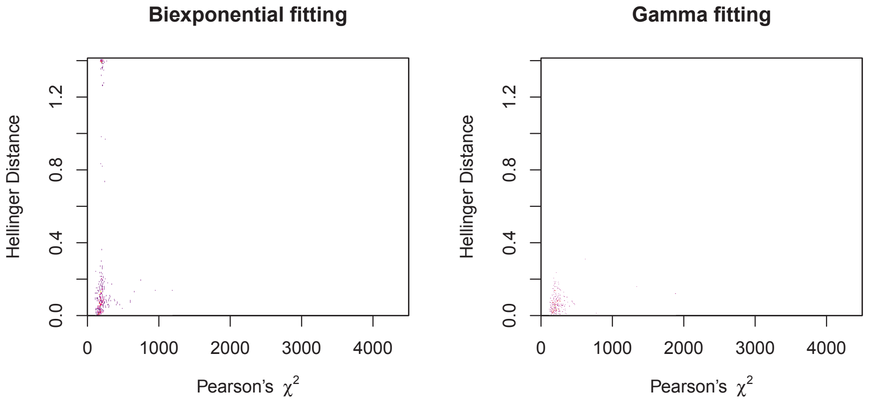

(b) $c = 0.75$, $k = 1.05$

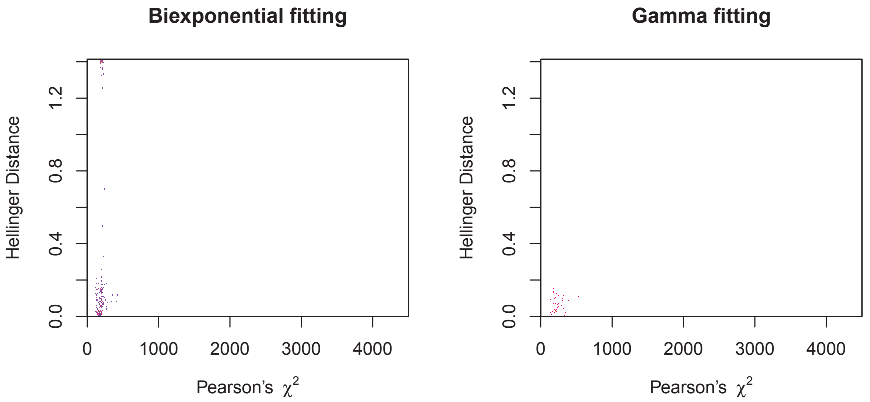

(c) $c = 0.75$, $k = 1.10$

Figure 11: Two-dimensional histogram for Hellinger Distance (vertical axis) and Pearson's χ^2 statistic (horizontal axis) for simulated data, where the plotted values have been aggregated over varying true values of c and τ_2. For each subfigure (a) through (c), fitting the biexponential directly gives the plot on the left, and using gamma conversion method gives the plot on the right. Intensity is graded from blue (lowest) to yellow (highest), white indicating no counts

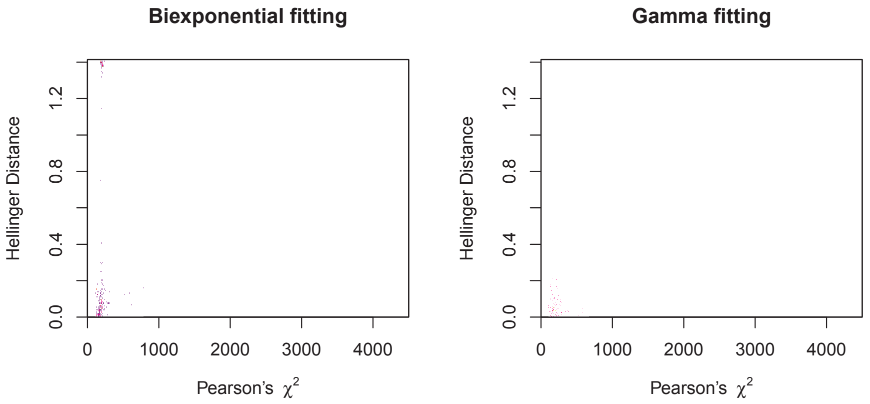

(a) $c = 0.75$, $k = 1.20$

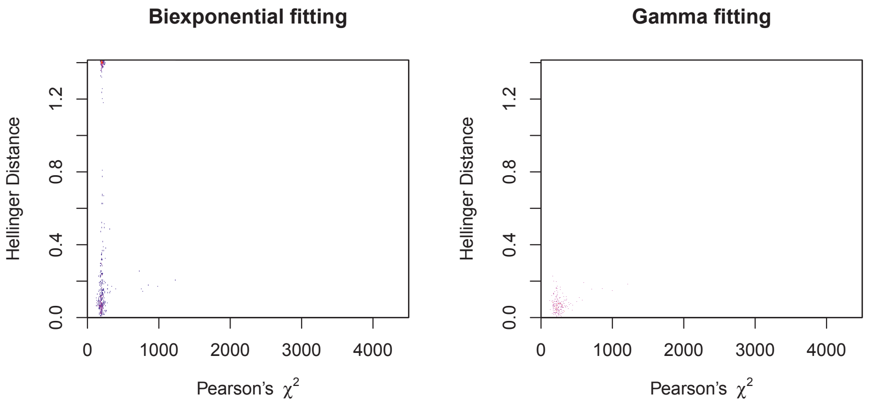

(b) $c = 0.75$, $k = 2.00$

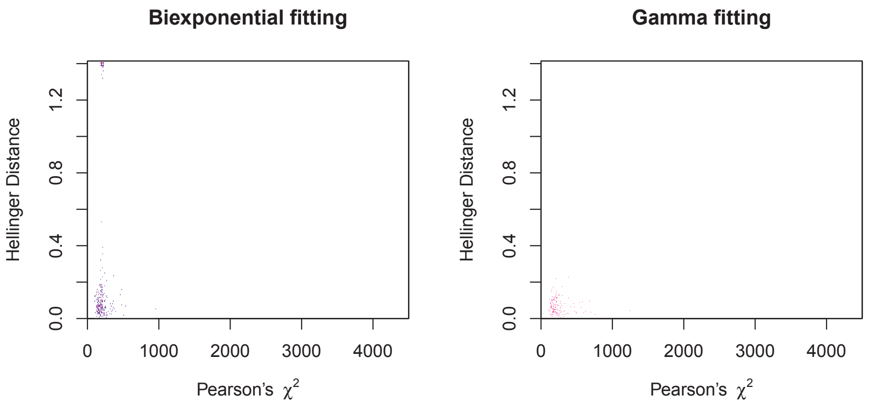

(c) $c = 0.90$, $k = 0.500$

Figure 12: Two-dimensional histogram for Hellinger Distance (vertical axis) and Pearson's χ^2 statistic (horizontal axis) for simulated data, where the plotted values have been aggregated over varying true values of c and τ_2. For each subfigure (a) through (c), fitting the biexponential directly gives the plot on the left, and using gamma conversion method gives the plot on the right. Intensity is graded from blue (lowest) to yellow (highest), white indicating no counts

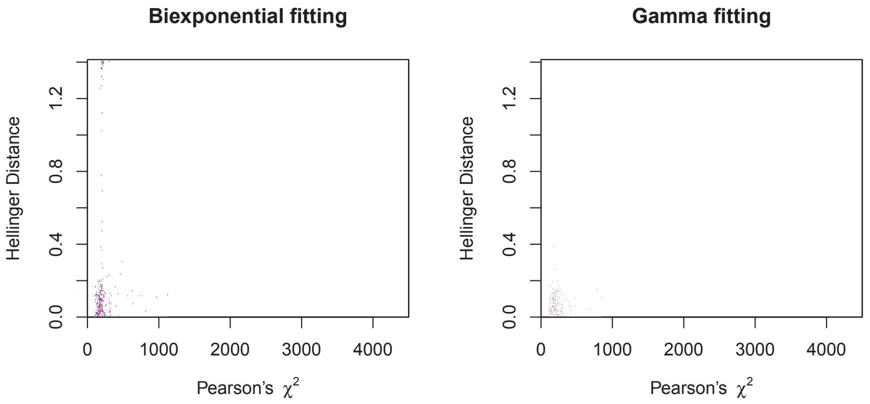

(a) $c = 0.90, k = 0.800$

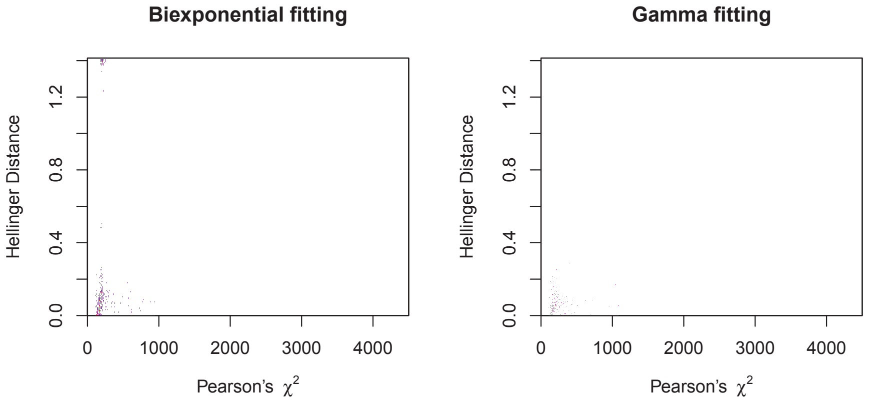

(b) $c = 0.90, k = 0.900$

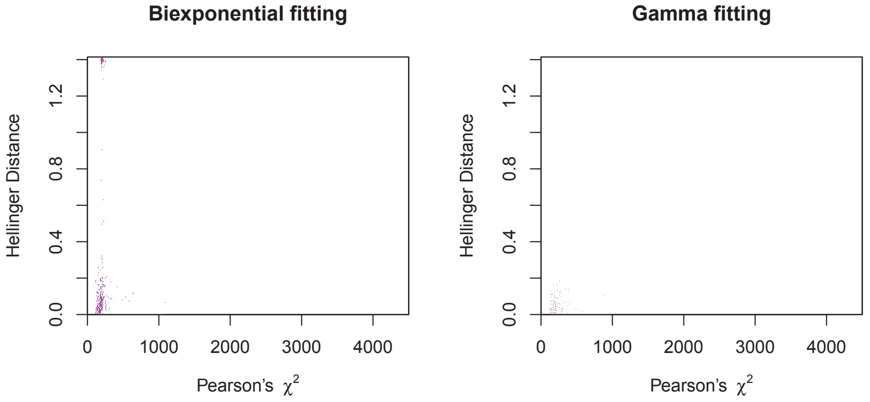

(c) $c = 0.90, k = 0.950$

Figure 13: Two-dimensional histogram for Hellinger Distance (vertical axis) and Pearson's χ^2 statistic (horizontal axis) for simulated data, where the plotted values have been aggregated over varying true values of c and τ_2. For each subfigure (a) through (c), fitting the biexponential directly gives the plot on the left, and using gamma conversion method gives the plot on the right. Intensity is graded from blue (lowest) to yellow (highest), white indicating no counts

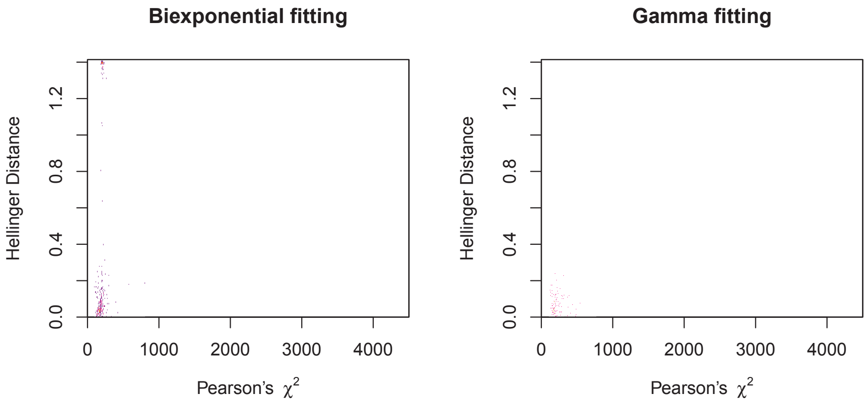

(a) $c = 0.90$, $k = 0.990$

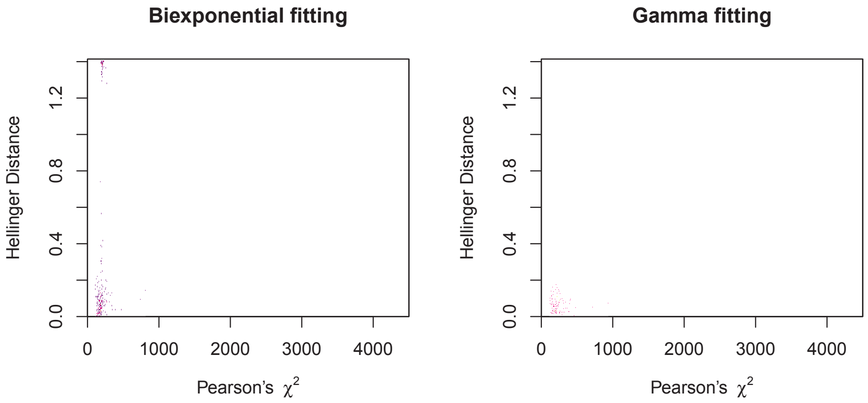

(b) $c = 0.90$, $k = 1.01$

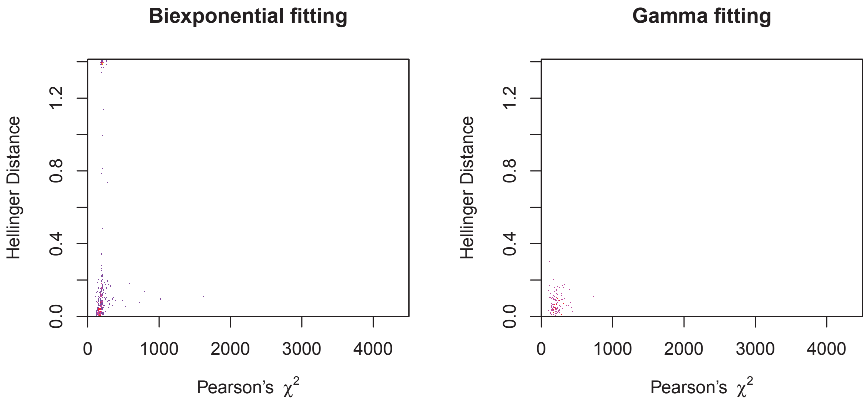

(c) $c = 0.90$, $k = 1.05$

Figure 14: Two-dimensional histogram for Hellinger Distance (vertical axis) and Pearson's χ^2 statistic (horizontal axis) for simulated data, where the plotted values have been aggregated over varying true values of c and τ_2. For each subfigure (a) through (c), fitting the biexponential directly gives the plot on the left, and using gamma conversion method gives the plot on the right. Intensity is graded from blue (lowest) to yellow (highest), white indicating no counts

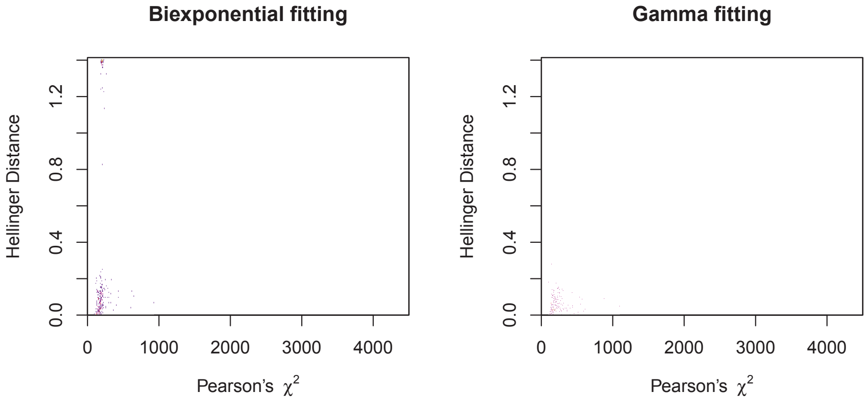

(a) $c = 0.90$, $k = 1.10$

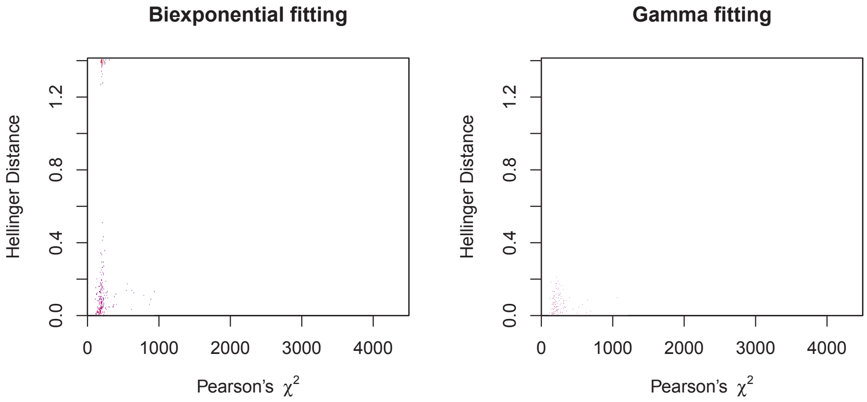

(b) $c = 0.90$, $k = 1.20$

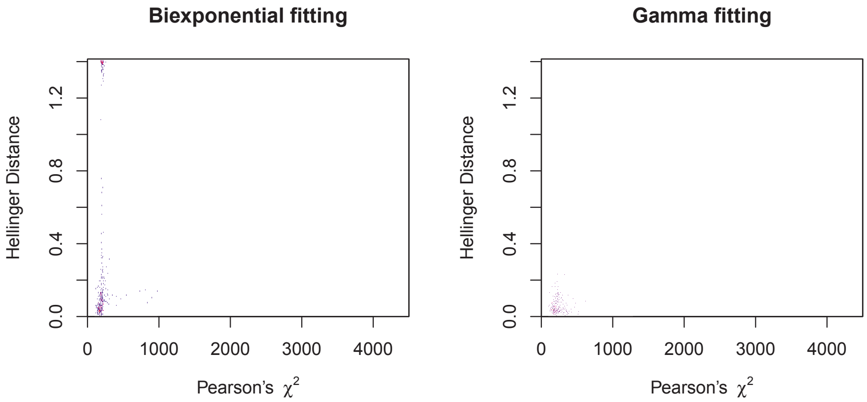

(c) $c = 0.90$, $k = 2.00$

Figure 15: Two-dimensional histogram for Hellinger Distance (vertical axis) and Pearson's χ^2 statistic (horizontal axis) for simulated data, where the plotted values have been aggregated over varying true values of c and τ_2. For each subfigure (a) through (c), fitting the biexponential directly gives the plot on the left, and using gamma conversion method gives the plot on the right. Intensity is graded from blue (lowest) to yellow (highest), white indicating no counts

Where τ_1 is not known

Here we consider the case where τ_1 is not assumed to be known. The same simulation data sets and real data sets as described in the paper were analyzed. We wish to compare how the estimations compared to the truth; to avoid identifiability issues, we present the estimated decay curves with the true curve superimposed. Figures 16 to 25 give these plots corresponding to fitting the biexponential directly and to the gamma conversion method for each of the thirty simulation configurations. Figure 26 gives this plot for the real data analysis. Besides getting a visualization on how well the estimation methods match the truth, we specifically wish to investigate the tendency to overfit the data. To this end, we again plot the Hellinger Distances, which gives a good idea as to how closely the estimated curves match with the truth, vs. Pearson's χ^2 statistics, which show how well the estimations fit the individual small data sets. Figure 27 gives the two-dimensional histogram of Hellinger Distances vs. χ^2 statistics aggregating over all 30 simulation configurations; Figure 28 gives the same for the real data sets. Figures 29 through 38 give these two-dimensional histograms separated by simulation configuration.

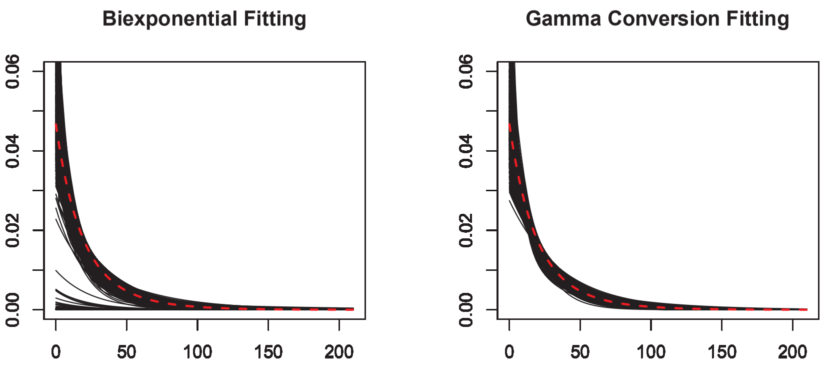

(a) $c = 0.6, k = 0.500$

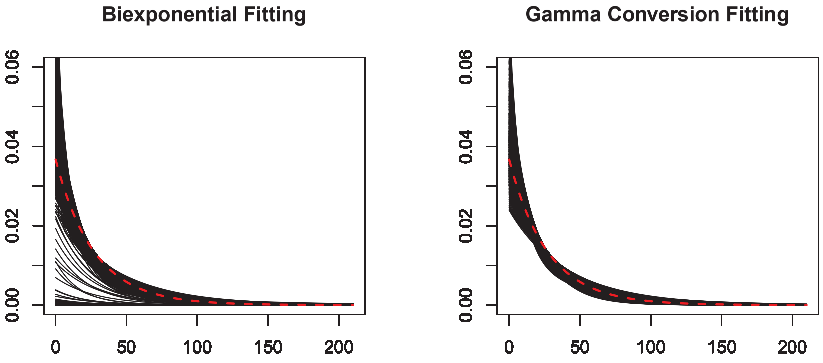

(b) $c = 0.6, k = 0.800$

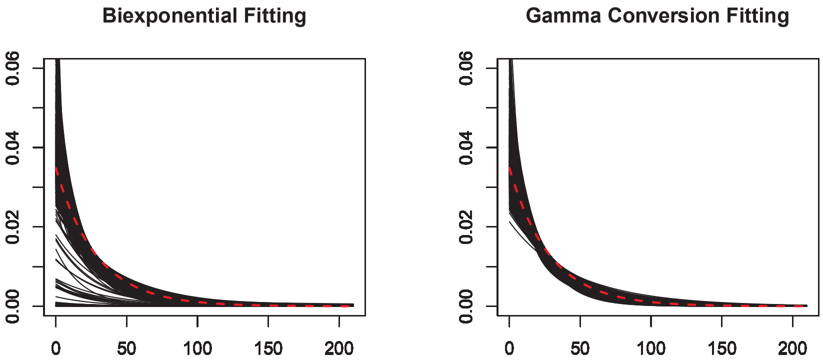

(c) $c = 0.6, k = 0.900$

Figure 16: Estimated decay curves for simulated data where τ_1 is unknown, with the true curve superimposed. For each subfigure (a) through (c), fitting the biexponential directly gives the plot on the left, and using gamma conversion method gives the plot on the right.

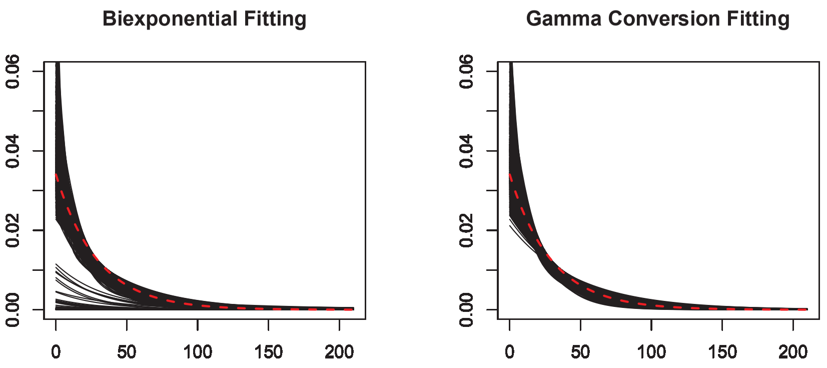

(a) $c = 0.6$, $k = 0.950$

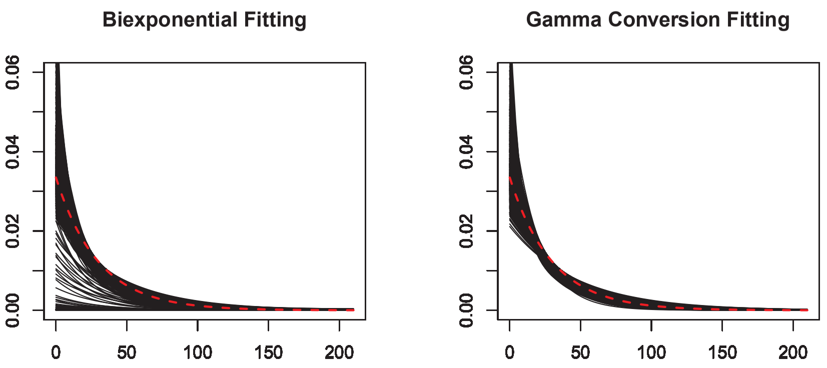

(b) $c = 0.6$, $k = 0.990$

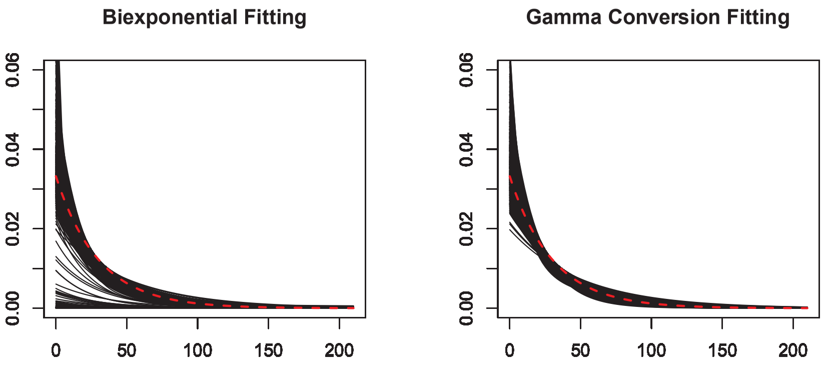

(c) $c = 0.6$, $k = 1.01$

Figure 17: Estimated decay curves for simulated data where τ_1 is unknown, with the true curve superimposed. For each subfigure (a) through (c), fitting the biexponential directly gives the plot on the left, and using gamma conversion method gives the plot on the right.
