# Supplementary material for: A parameter estimation method for fluorescence lifetime data
Source: BMC Res Notes. 2015 Jun 9;8:230. doi: 10.1186/s13104-015-1176-y (PMC4467687; doi:10.1186/s13104-015-1176-y)
Supplement: Supplementary file 2 — Additional file 2: Numerical results 2 [file 13104_2015_1176_MOESM2_ESM.pdf]

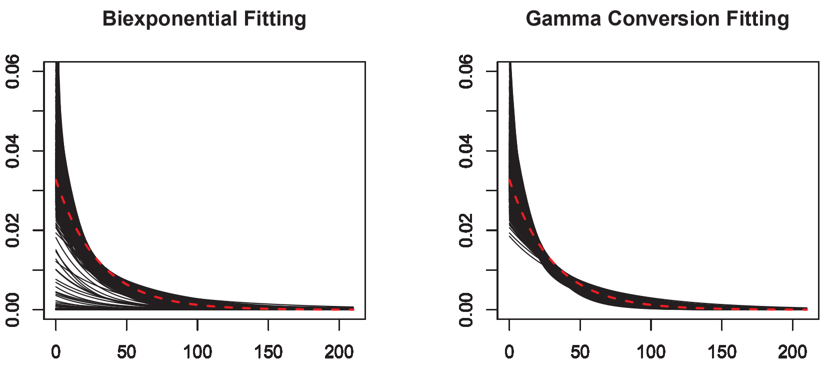

(a) $c = 0.6, k = 1.05$

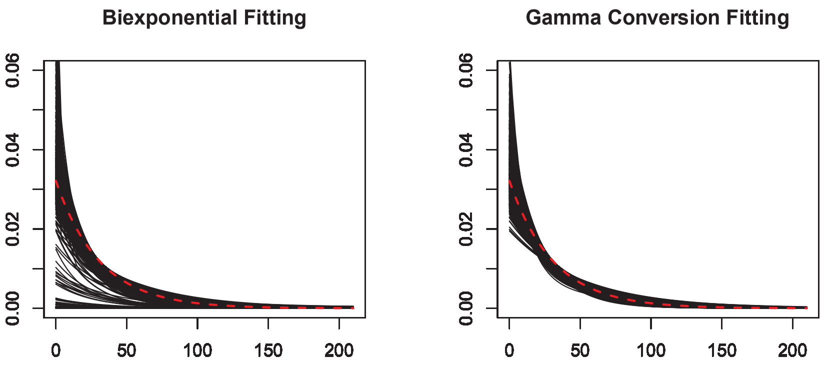

(b) $c = 0.6, k = 1.10$

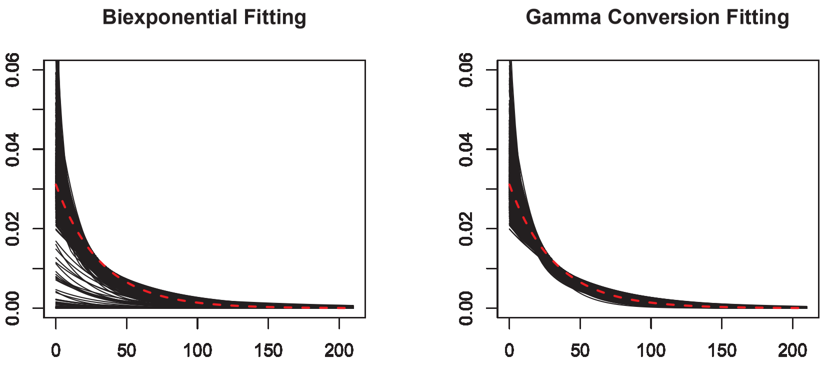

(c) $c = 0.6, k = 1.20$

Figure 18: Estimated decay curves for simulated data where τ_1 is unknown, with the true curve superimposed. For each subfigure (a) through (c), fitting the biexponential directly gives the plot on the left, and using gamma conversion method gives the plot on the right.

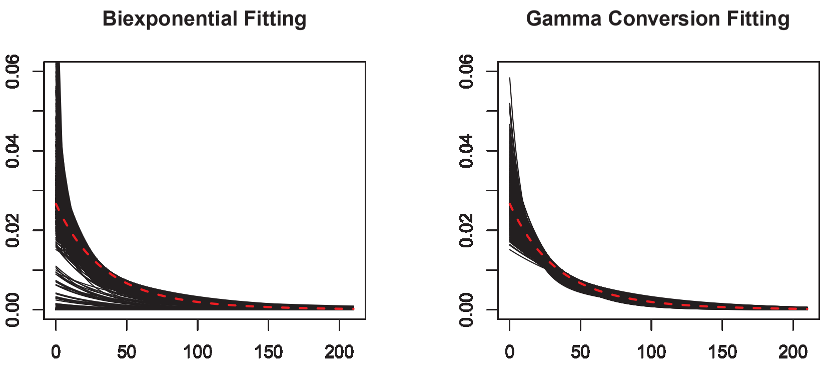

(a) $c = 0.6$, $k = 2.00$

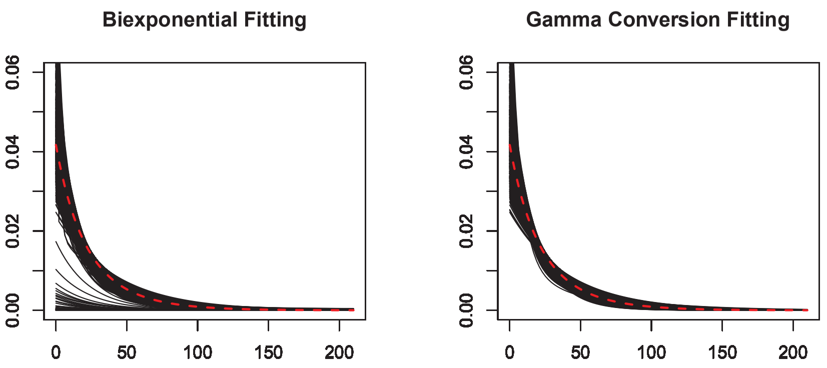

(b) $c = 0.75$, $k = 0.500$

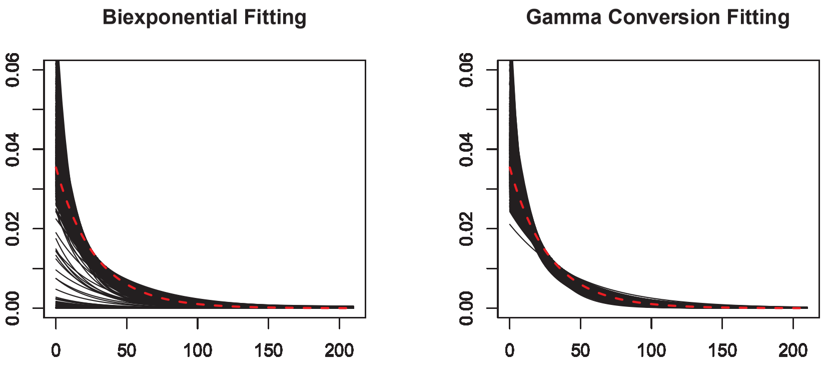

(c) $c = 0.75$, $k = 0.800$

Figure 19: Estimated decay curves for simulated data where τ_1 is unknown, with the true curve superimposed. For each subfigure (a) through (c), fitting the biexponential directly gives the plot on the left, and using gamma conversion method gives the plot on the right.

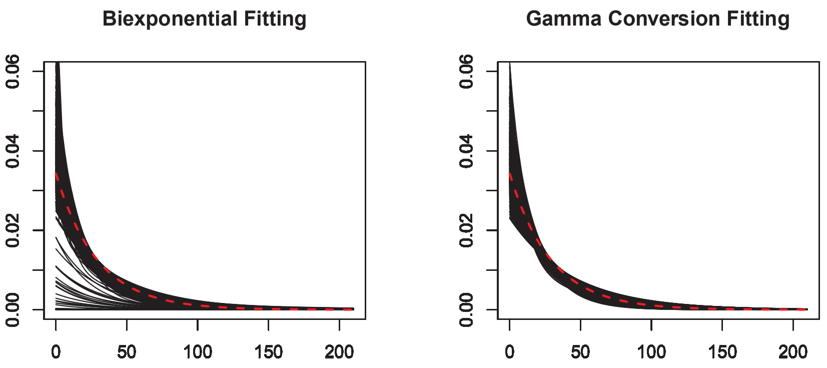

(a) $c = 0.75, k = 0.900$

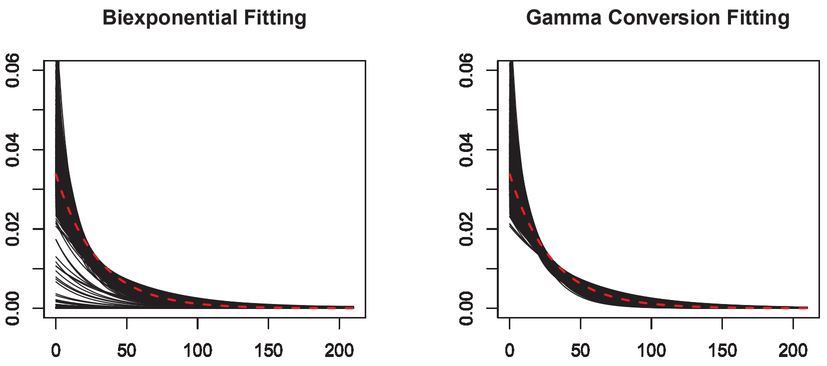

(b) $c = 0.75, k = 0.950$

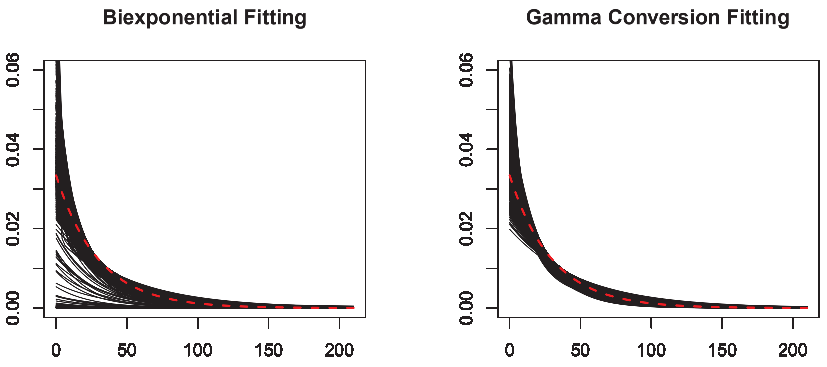

(c) $c = 0.75, k = 0.990$

Figure 20: Estimated decay curves for simulated data where τ_1 is unknown, with the true curve superimposed. For each subfigure (a) through (c), fitting the biexponential directly gives the plot on the left, and using gamma conversion method gives the plot on the right.

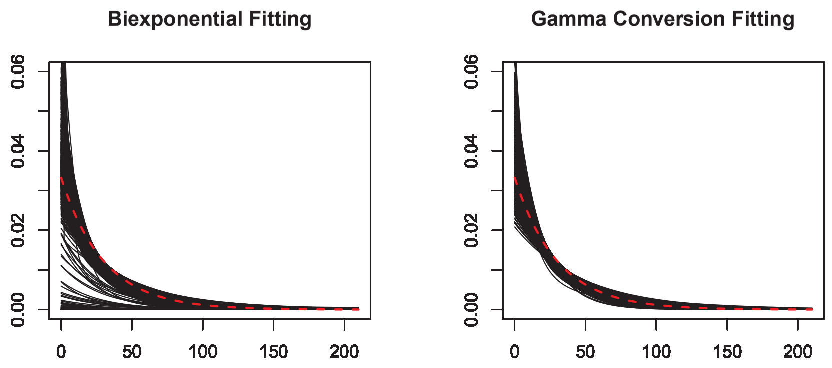

(a) $c = 0.75, k = 1.01$

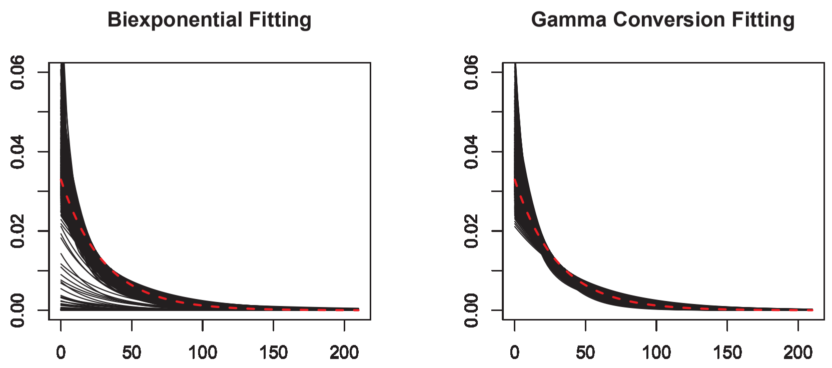

(b) $c = 0.75, k = 1.05$

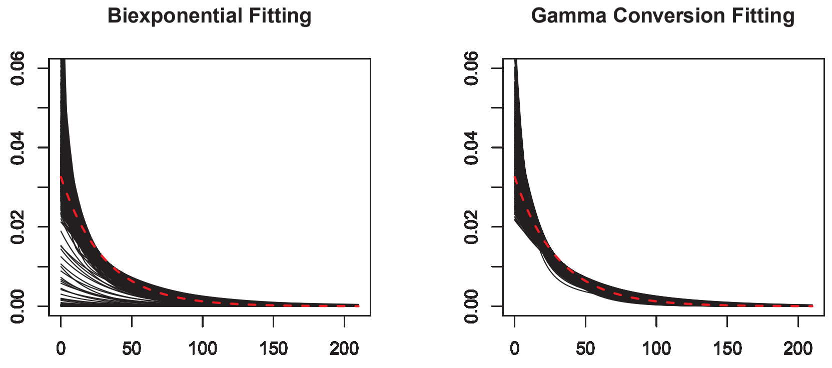

(c) $c = 0.75, k = 1.10$

Figure 21: Estimated decay curves for simulated data where τ_1 is unknown, with the true curve superimposed. For each subfigure (a) through (c), fitting the biexponential directly gives the plot on the left, and using gamma conversion method gives the plot on the right.

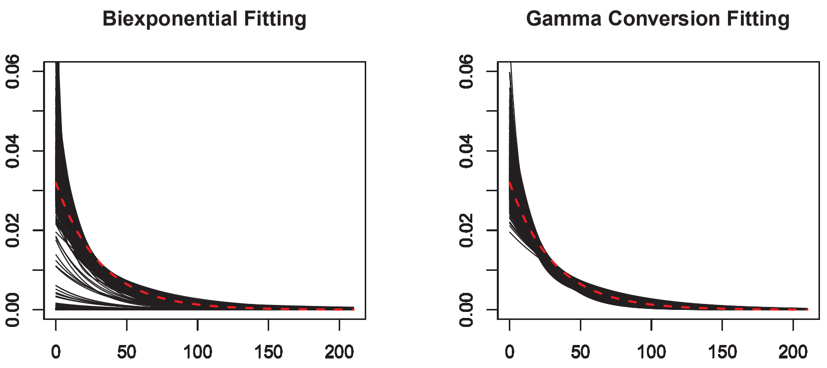

(a) $c = 0.75, k = 1.20$

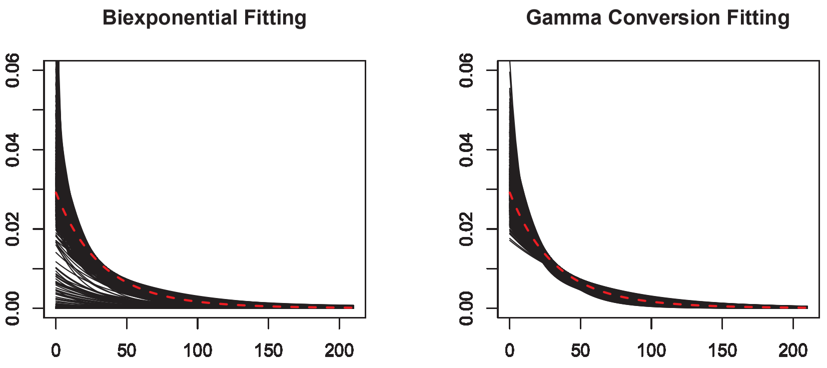

(b) $c = 0.75, k = 2.00$

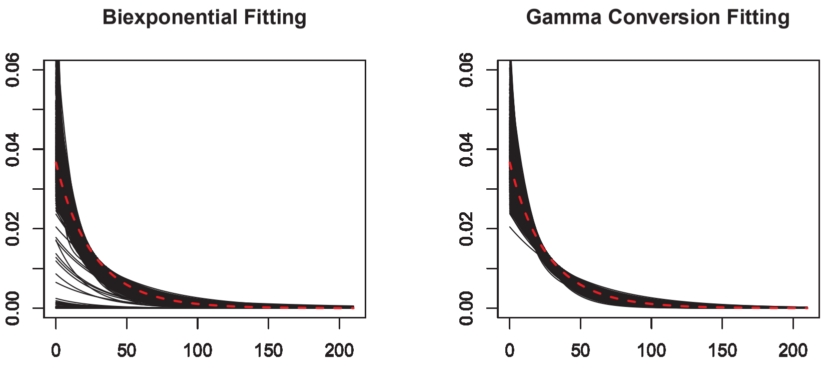

(c) $c = 0.90, k = 0.500$

Figure 22: Estimated decay curves for simulated data where τ_1 is unknown, with the true curve superimposed. For each subfigure (a) through (c), fitting the biexponential directly gives the plot on the left, and using gamma conversion method gives the plot on the right.
