# Supplementary material for: A parameter estimation method for fluorescence lifetime data
Source: BMC Res Notes. 2015 Jun 9;8:230. doi: 10.1186/s13104-015-1176-y (PMC4467687; doi:10.1186/s13104-015-1176-y)
Supplement: Supplementary file 3 — Additional file 3: Numerical results 3 [file 13104_2015_1176_MOESM3_ESM.pdf]

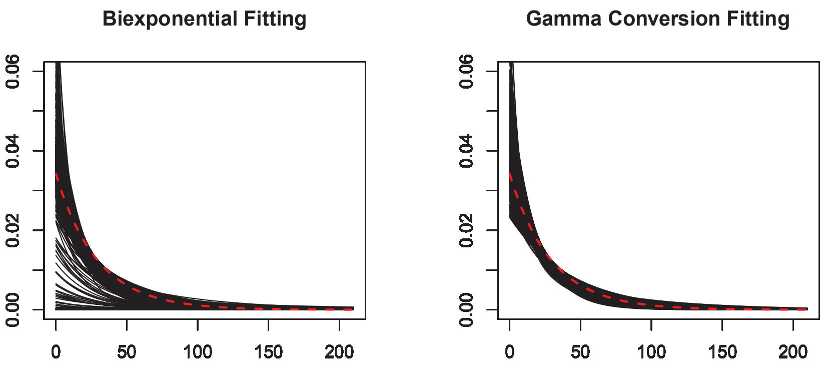

(a) $c = 0.90, k = 0.800$

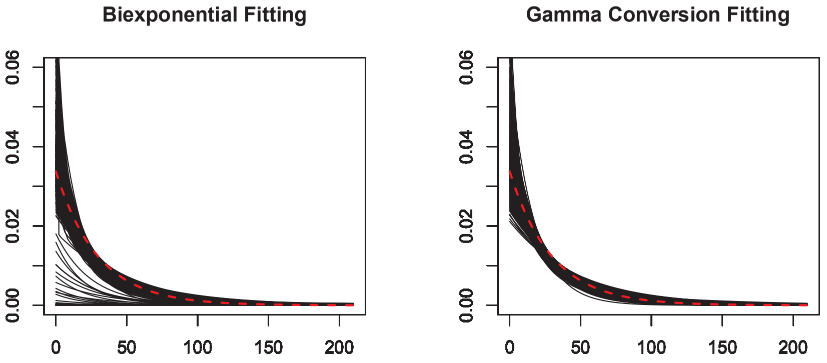

(b) $c = 0.90, k = 0.900$

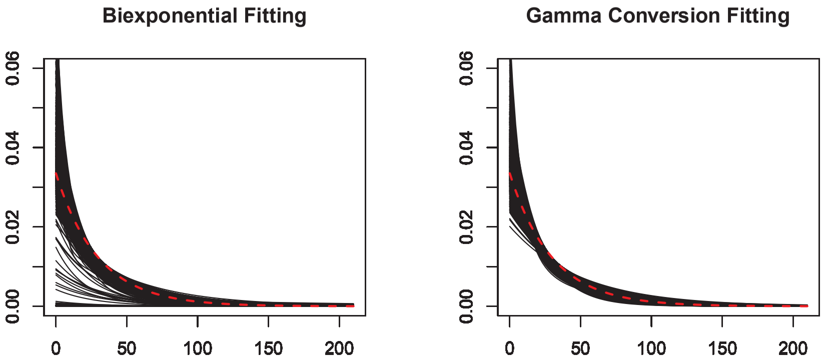

(c) $c = 0.90, k = 0.950$

Figure 23: Estimated decay curves for simulated data where τ_1 is unknown, with the true curve superimposed. For each subfigure (a) through (c), fitting the biexponential directly gives the plot on the left, and using gamma conversion method gives the plot on the right.

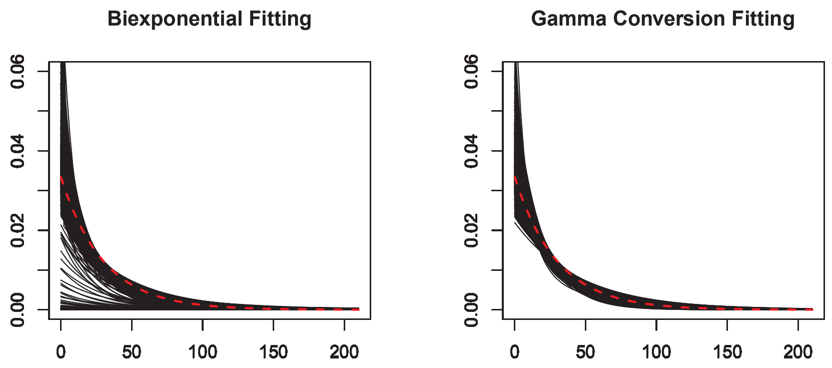

(a) $c = 0.90, k = 0.990$

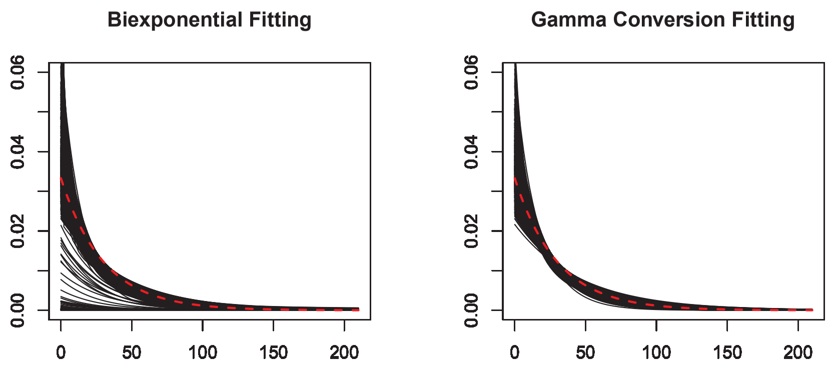

(b) $c = 0.90, k = 1.01$

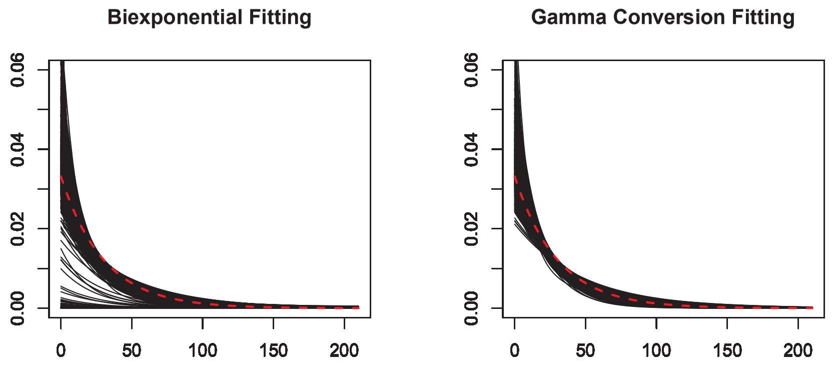

(c) $c = 0.90, k = 1.05$

Figure 24: Estimated decay curves for simulated data where τ_1 is unknown, with the true curve superimposed. For each subfigure (a) through (c), fitting the biexponential directly gives the plot on the left, and using gamma conversion method gives the plot on the right.

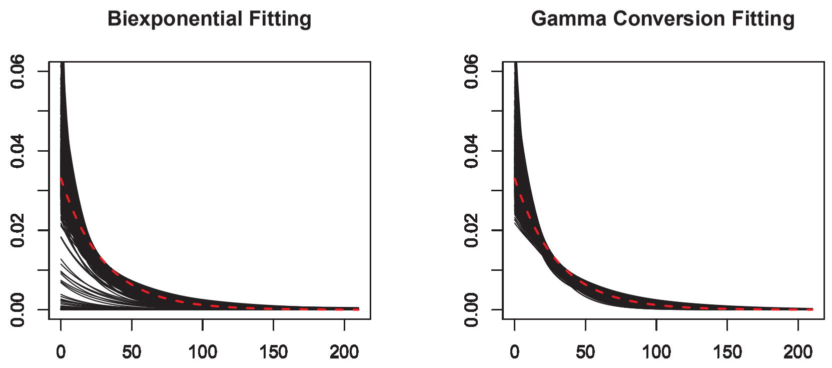

(a) $c = 0.90, k = 1.10$

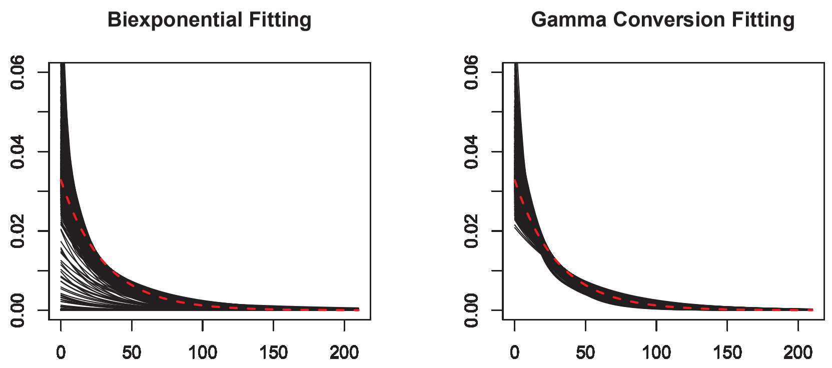

(b) $c = 0.90, k = 1.20$

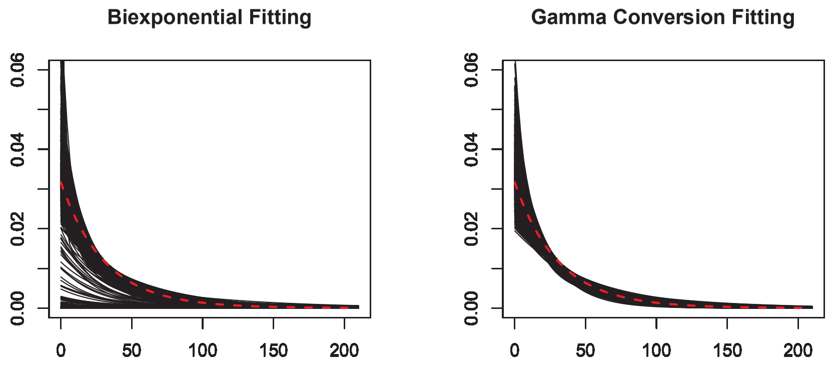

(c) $c = 0.90, k = 2.00$

Figure 25: Estimated decay curves for simulated data where τ_1 is unknown, with the true curve superimposed. For each subfigure (a) through (c), fitting the biexponential directly gives the plot on the left, and using gamma conversion method gives the plot on the right.

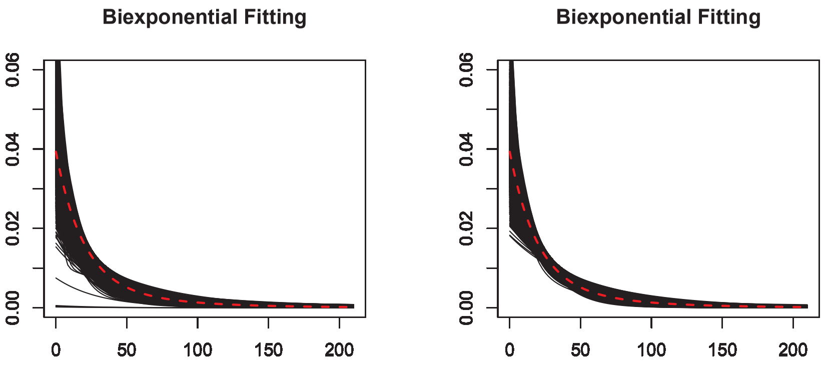

Figure 26: Estimated decay curves for fluorophore Cy3 data where τ_1 is unknown, with the true curve superimposed. Fitting the biexponential directly gives the plot on the left, and using gamma conversion method gives the plot on the right.

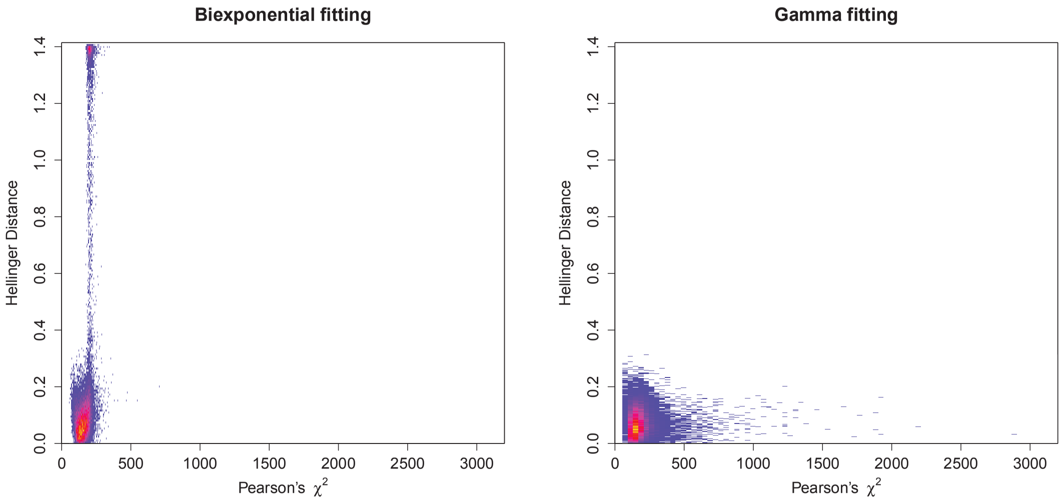

(a) (b)

Figure 27: Two-dimensional histogram for Hellinger Distance (vertical axis) and Pearson's χ^2 statistic (horizontal axis) for simulated data with τ_1 unknown, where the plotted values have been aggregated over varying true values of c and τ_2. Fitting the biexponential directly gives the plot on the left, and using gamma conversion method gives the plot on the right. Intensity is graded from blue (lowest) to yellow (highest), white indicating no counts

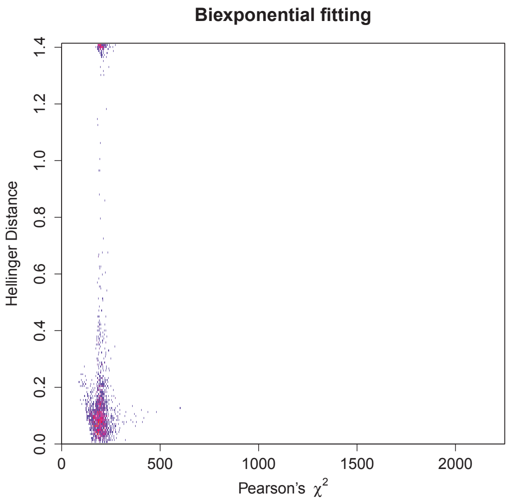

(a)

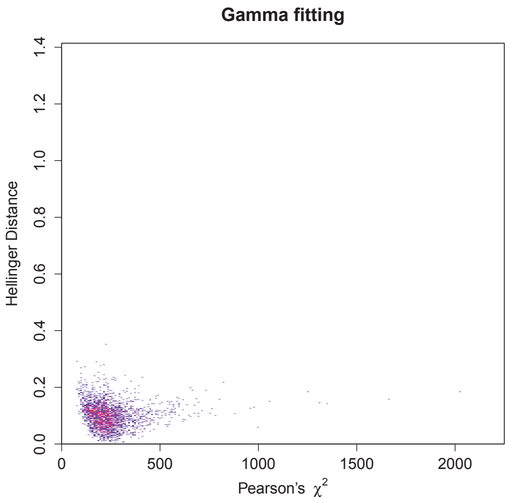

(b)

Figure 28: Two-dimensional histogram for Hellinger Distance (vertical axis) and Pearson's χ^2 statistic (horizontal axis) for fluorophore Cy3 data with τ_1 unknown. Fitting the biexponential directly gives the plot on the left, and using gamma conversion method gives the plot on the right. Intensity is graded from blue (lowest) to yellow (highest), white indicating no counts
