# Supplementary material for: A parameter estimation method for fluorescence lifetime data
Source: BMC Res Notes. 2015 Jun 9;8:230. doi: 10.1186/s13104-015-1176-y (PMC4467687; doi:10.1186/s13104-015-1176-y)
Supplement: Supplementary file 4 — Additional file 4: Numerical results 4 [file 13104_2015_1176_MOESM4_ESM.pdf]

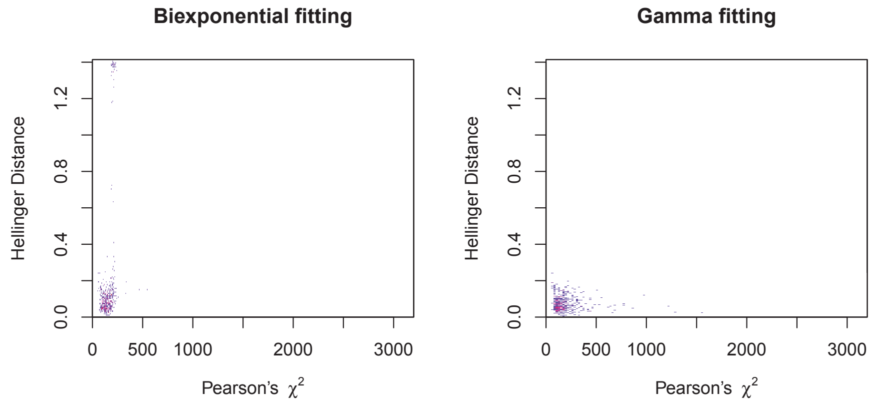

(a) $c = 0.6$, $k = 0.500$

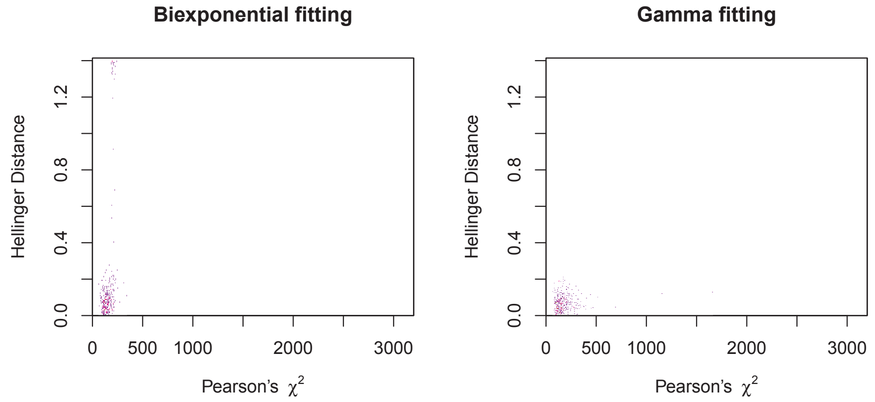

(b) $c = 0.6$, $k = 0.800$

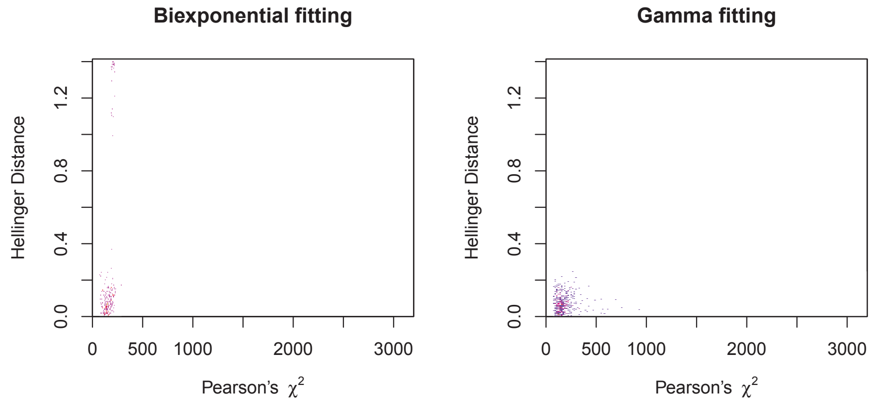

(c) $c = 0.6$, $k = 0.900$

Figure 29: Two-dimensional histogram for Hellinger Distance (vertical axis) and Pearson's χ^2 statistic (horizontal axis) for simulated data, where the plotted values have been aggregated over varying true values of c and τ_2. For each subfigure (a) through (c), fitting the biexponential directly gives the plot on the left, and using gamma conversion method gives the plot on the right. Intensity is graded from blue (lowest) to yellow (highest), white indicating no counts

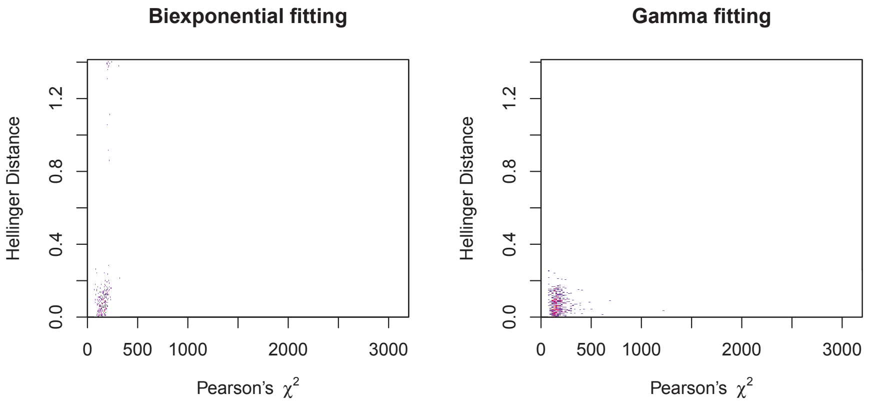

(a) $c = 0.6$, $k = 0.950$

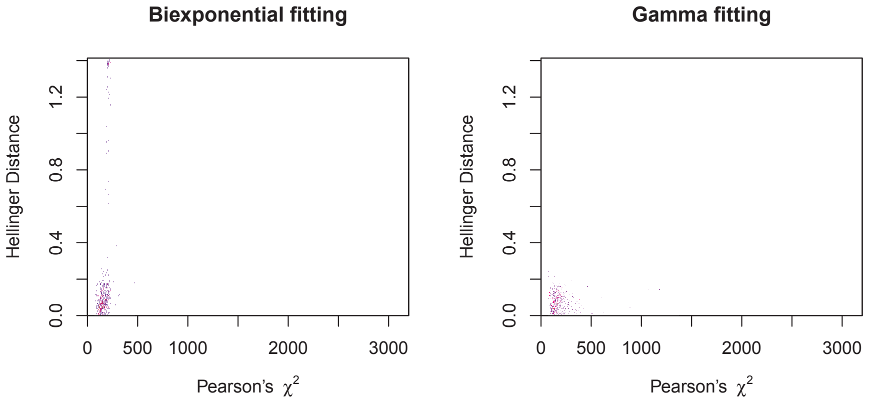

(b) $c = 0.6$, $k = 0.990$

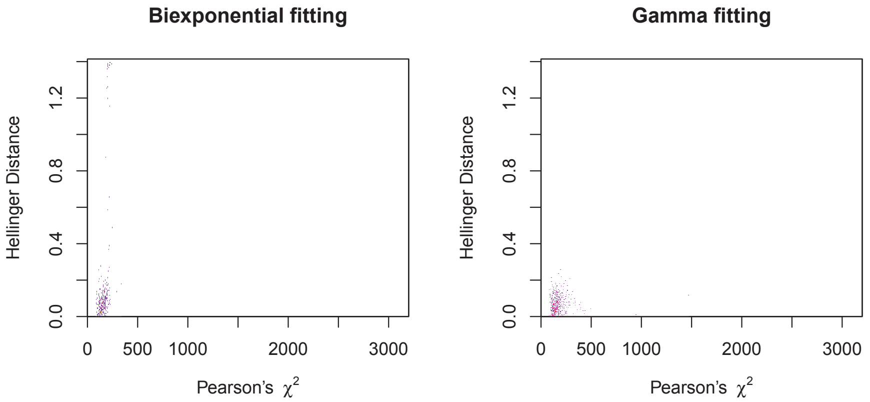

(c) $c = 0.6$, $k = 1.01$

Figure 30: Two-dimensional histogram for Hellinger Distance (vertical axis) and Pearson's χ^2 statistic (horizontal axis) for simulated data, where the plotted values have been aggregated over varying true values of c and τ_2. For each subfigure (a) through (c), fitting the biexponential directly gives the plot on the left, and using gamma conversion method gives the plot on the right. Intensity is graded from blue (lowest) to yellow (highest), white indicating no counts

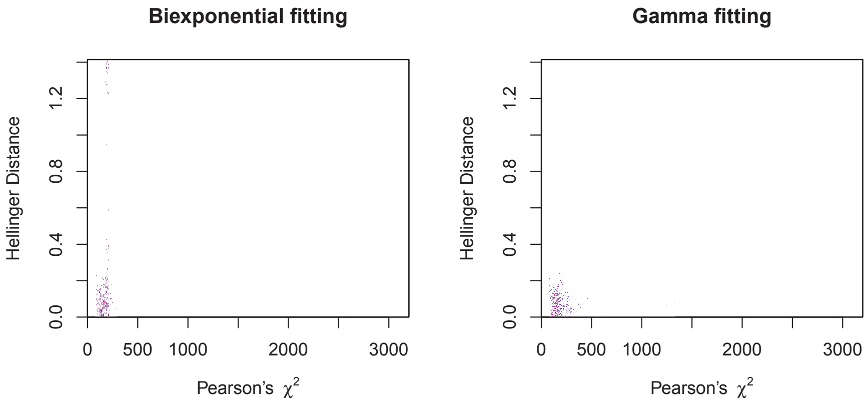

(a) $c = 0.6$, $k = 1.05$

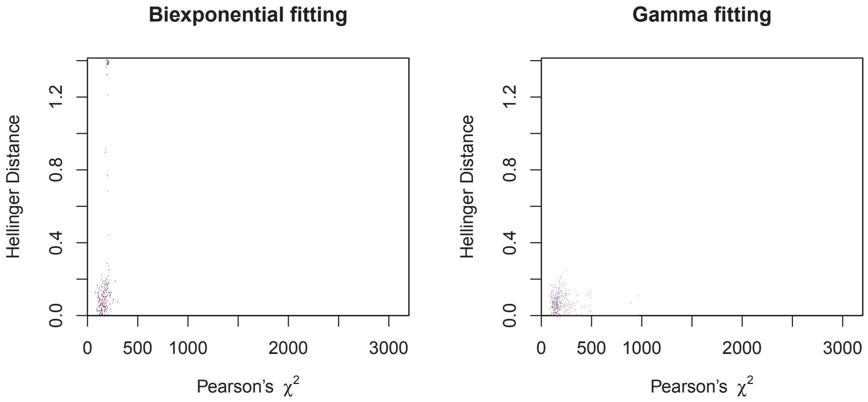

(b) $c = 0.6$, $k = 1.10$

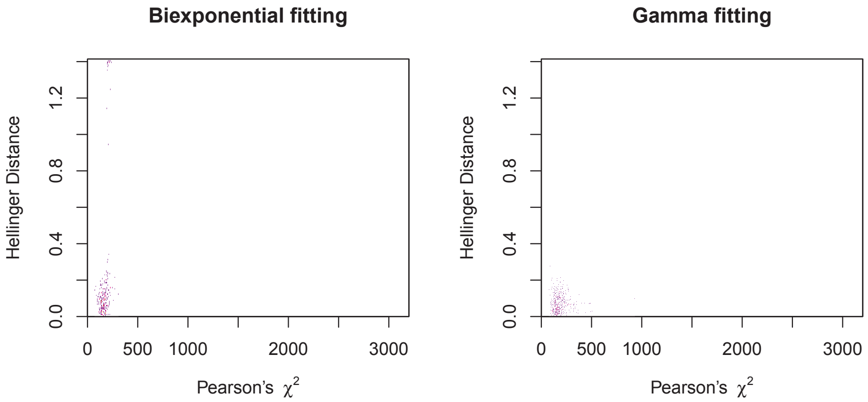

(c) $c = 0.6$, $k = 1.20$

Figure 31: Two-dimensional histogram for Hellinger Distance (vertical axis) and Pearson's χ^2 statistic (horizontal axis) for simulated data, where the plotted values have been aggregated over varying true values of c and τ_2. For each subfigure (a) through (c), fitting the biexponential directly gives the plot on the left, and using gamma conversion method gives the plot on the right. Intensity is graded from blue (lowest) to yellow (highest), white indicating no counts

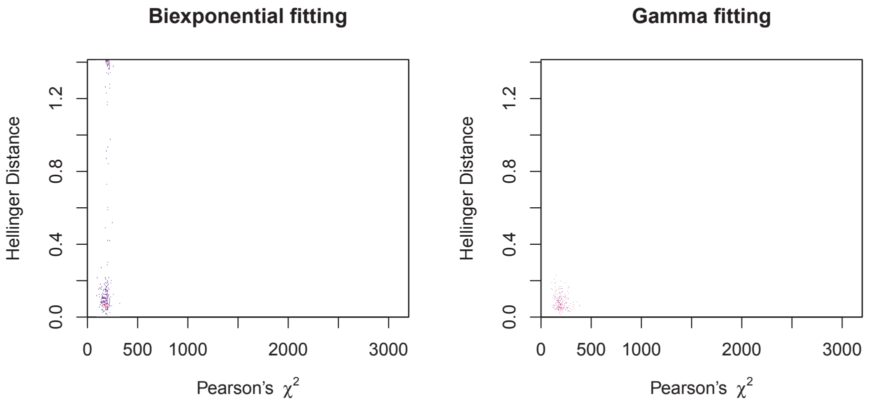

(a) $c = 0.6$, $k = 2.00$

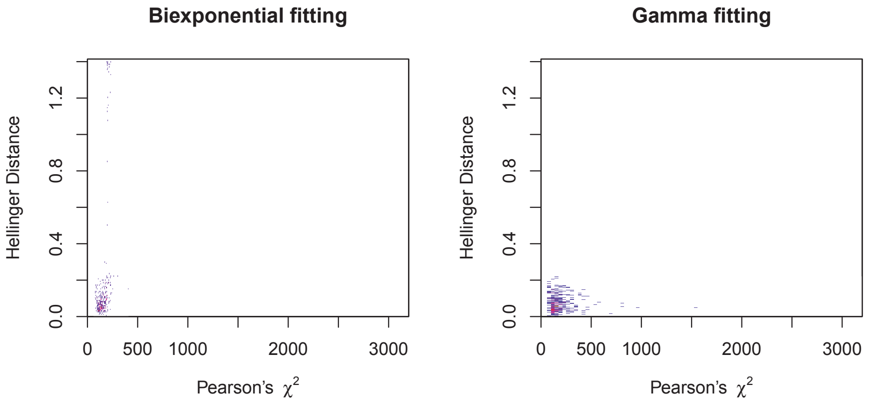

(b) $c = 0.75$, $k = 0.500$

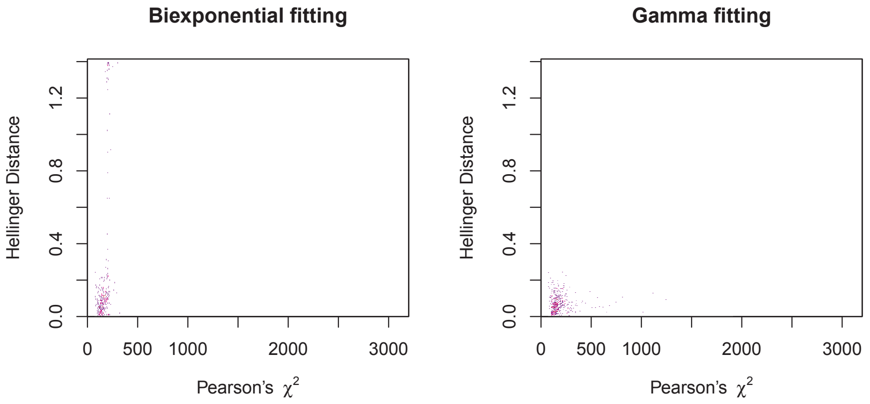

(c) $c = 0.75$, $k = 0.800$

Figure 32: Two-dimensional histogram for Hellinger Distance (vertical axis) and Pearson's χ^2 statistic (horizontal axis) for simulated data, where the plotted values have been aggregated over varying true values of c and τ_2. For each subfigure (a) through (c), fitting the biexponential directly gives the plot on the left, and using gamma conversion method gives the plot on the right. Intensity is graded from blue (lowest) to yellow (highest), white indicating no counts

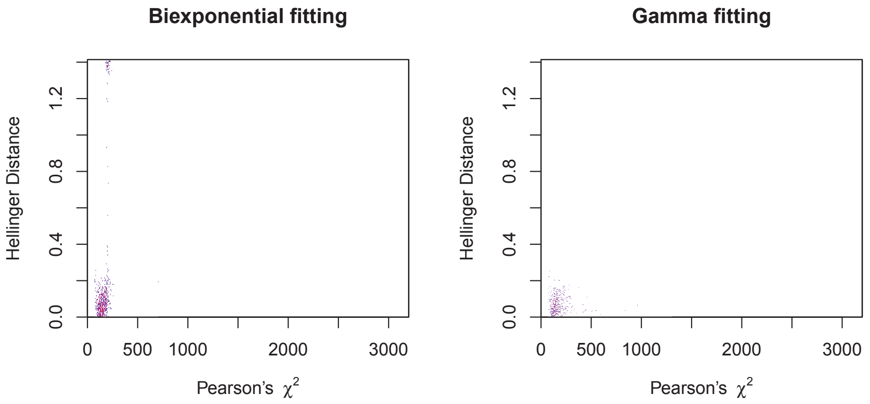

(a) $c = 0.75$, $k = 0.900$

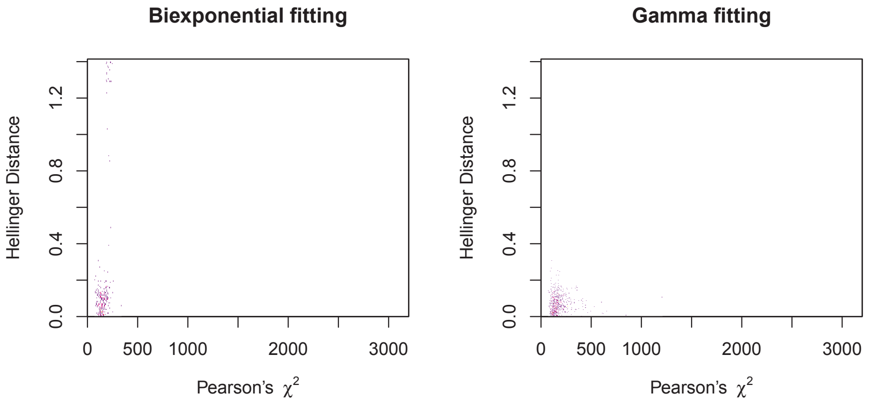

(b) $c = 0.75$, $k = 0.950$

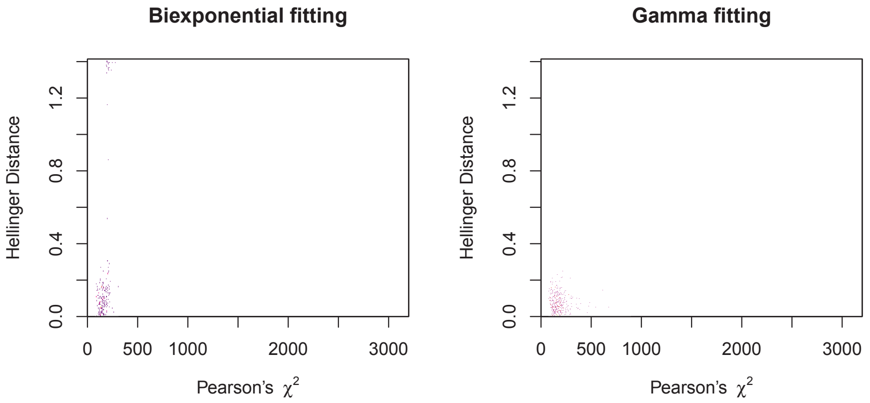

(c) $c = 0.75$, $k = 0.990$

Figure 33: Two-dimensional histogram for Hellinger Distance (vertical axis) and Pearson's χ^2 statistic (horizontal axis) for simulated data, where the plotted values have been aggregated over varying true values of c and τ_2. For each subfigure (a) through (c), fitting the biexponential directly gives the plot on the left, and using gamma conversion method gives the plot on the right. Intensity is graded from blue (lowest) to yellow (highest), white indicating no counts

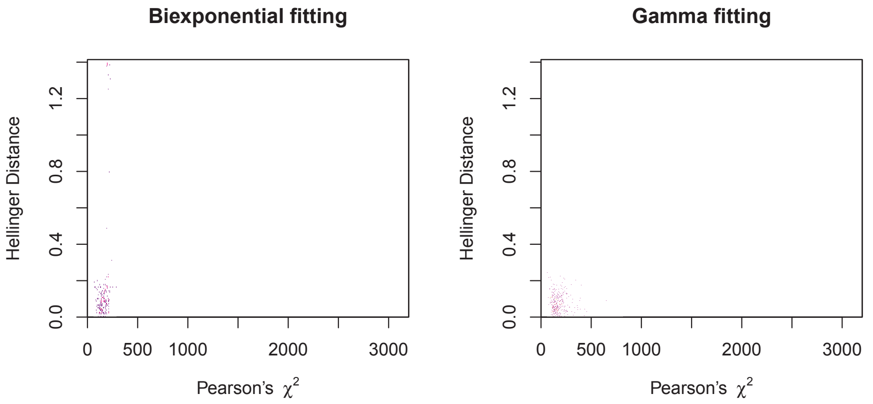

(a) $c = 0.75, k = 1.01$

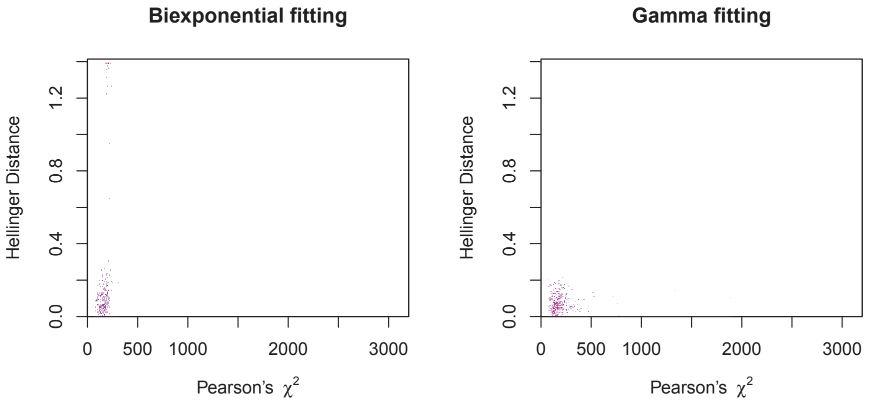

(b) $c = 0.75, k = 1.05$

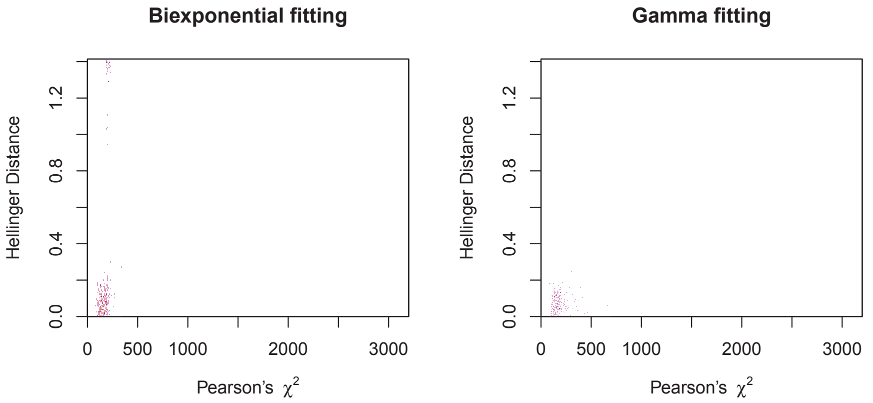

(c) $c = 0.75, k = 1.10$

Figure 34: Two-dimensional histogram for Hellinger Distance (vertical axis) and Pearson's χ^2 statistic (horizontal axis) for simulated data, where the plotted values have been aggregated over varying true values of c and τ_2. For each subfigure (a) through (c), fitting the biexponential directly gives the plot on the left, and using gamma conversion method gives the plot on the right. Intensity is graded from blue (lowest) to yellow (highest), white indicating no counts

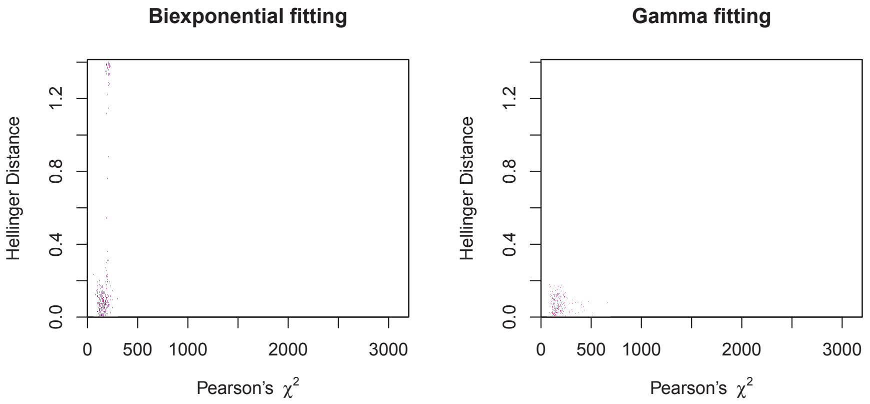

(a) $c = 0.75$, $k = 1.20$

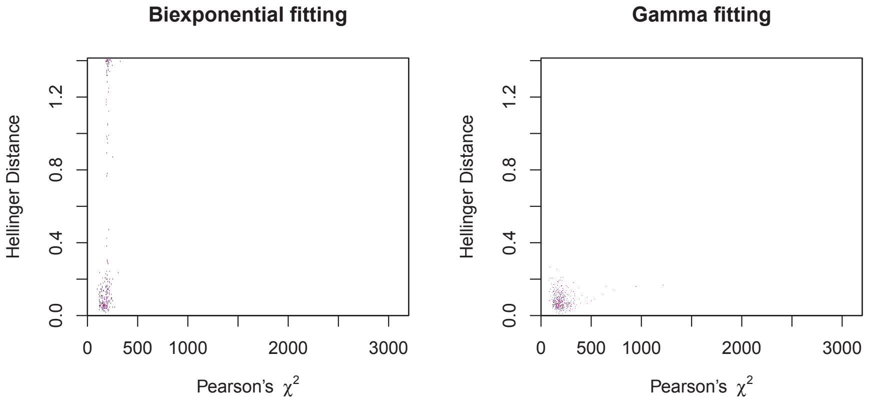

(b) $c = 0.75$, $k = 2.00$

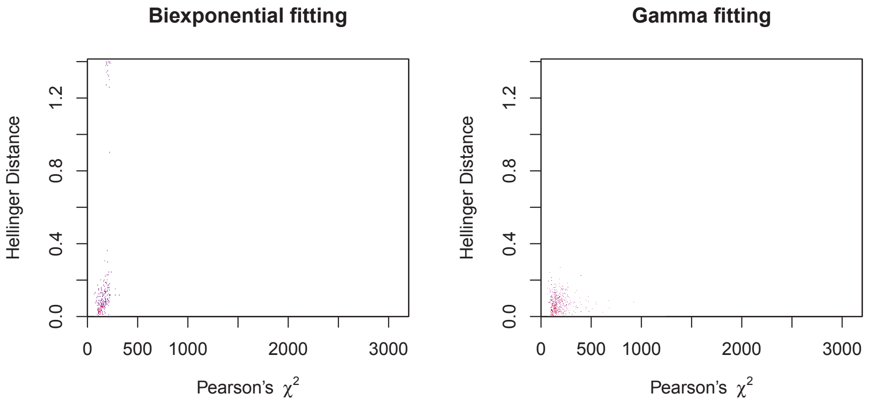

(c) $c = 0.90$, $k = 0.500$

Figure 35: Two-dimensional histogram for Hellinger Distance (vertical axis) and Pearson's χ^2 statistic (horizontal axis) for simulated data, where the plotted values have been aggregated over varying true values of c and τ_2. For each subfigure (a) through (c), fitting the biexponential directly gives the plot on the left, and using gamma conversion method gives the plot on the right. Intensity is graded from blue (lowest) to yellow (highest), white indicating no counts

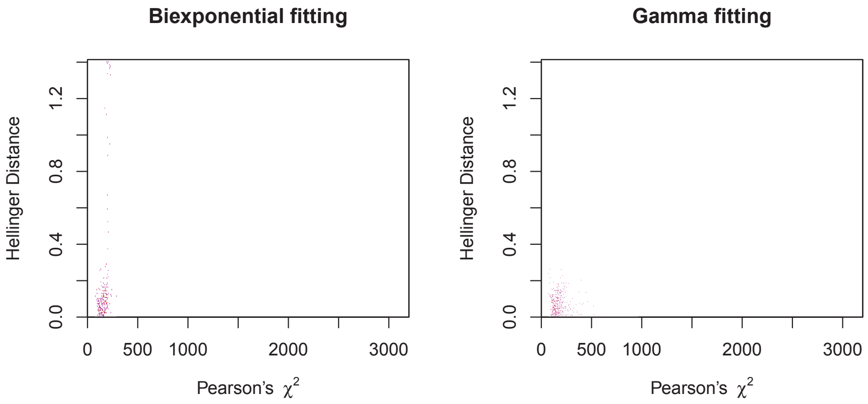

(a) $c = 0.90, k = 0.800$

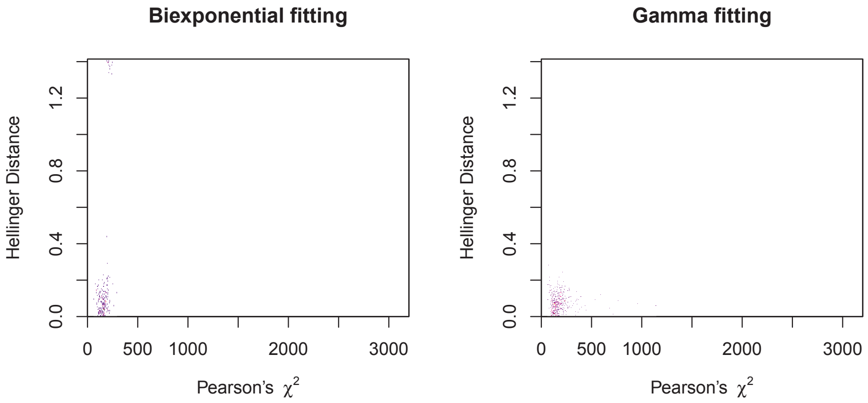

(b) $c = 0.90, k = 0.900$

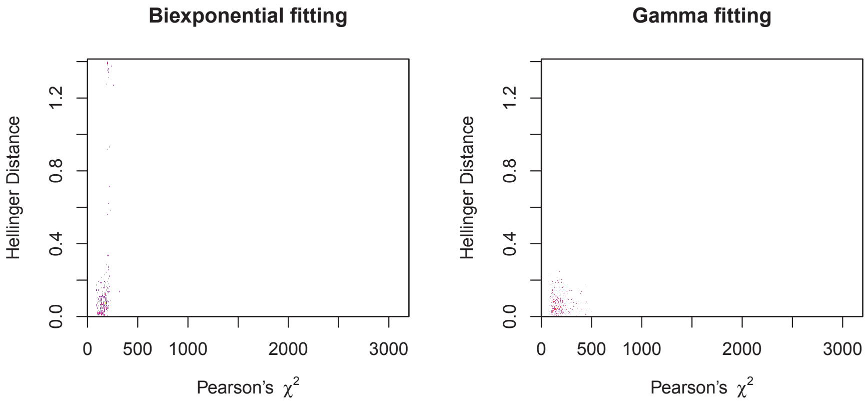

(c) $c = 0.90, k = 0.950$

Figure 36: Two-dimensional histogram for Hellinger Distance (vertical axis) and Pearson's χ^2 statistic (horizontal axis) for simulated data, where the plotted values have been aggregated over varying true values of c and τ_2. For each subfigure (a) through (c), fitting the biexponential directly gives the plot on the left, and using gamma conversion method gives the plot on the right. Intensity is graded from blue (lowest) to yellow (highest), white indicating no counts

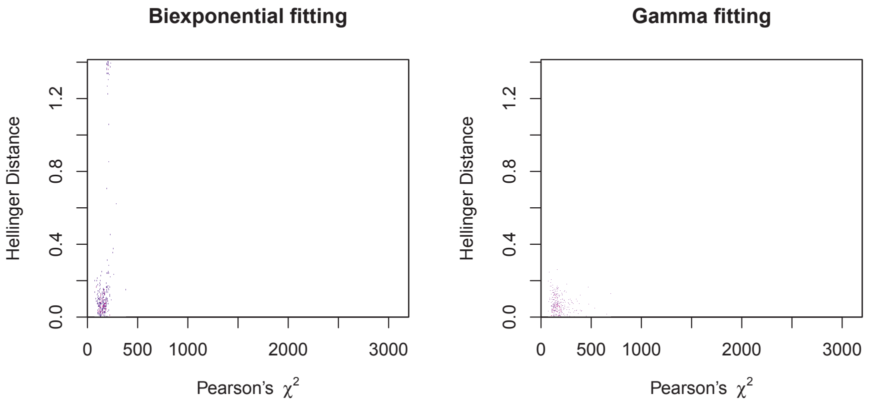

(a) $c = 0.90, k = 0.990$

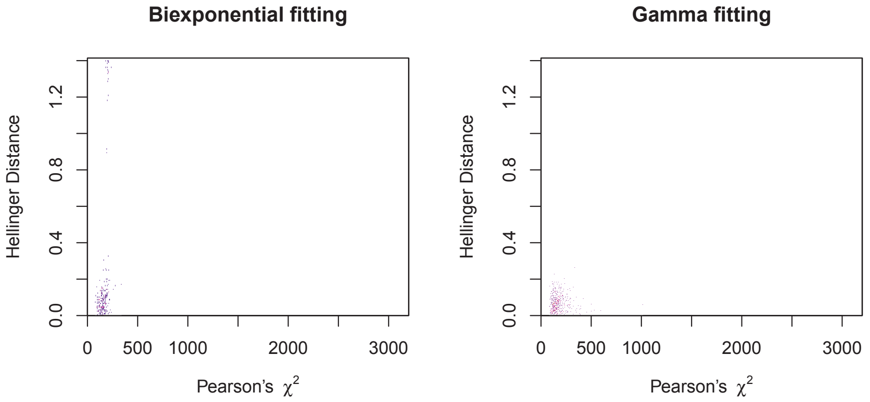

(b) $c = 0.90, k = 1.01$

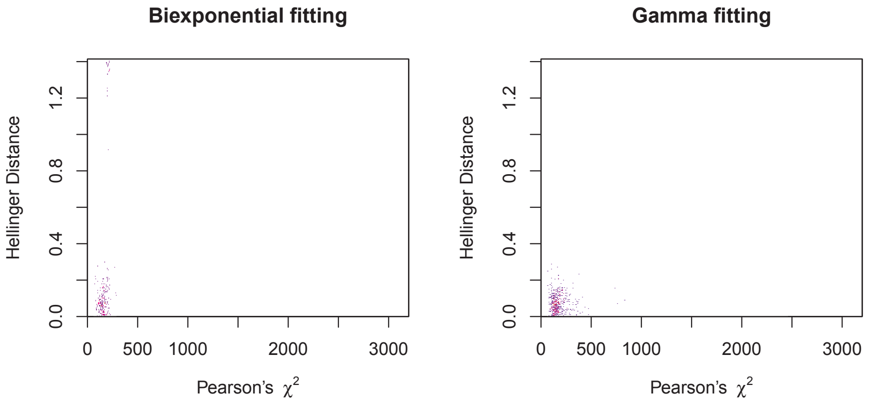

(c) $c = 0.90, k = 1.05$

Figure 37: Two-dimensional histogram for Hellinger Distance (vertical axis) and Pearson's χ^2 statistic (horizontal axis) for simulated data, where the plotted values have been aggregated over varying true values of c and τ_2. For each subfigure (a) through (c), fitting the biexponential directly gives the plot on the left, and using gamma conversion method gives the plot on the right. Intensity is graded from blue (lowest) to yellow (highest), white indicating no counts

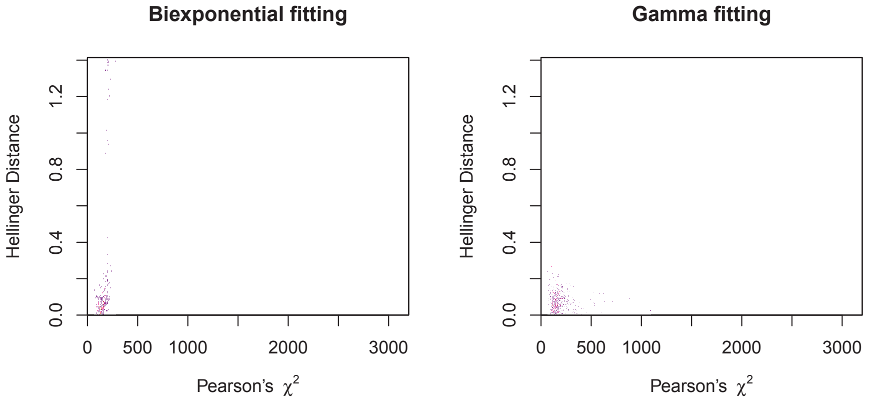

(a) $c = 0.90$, $k = 1.10$

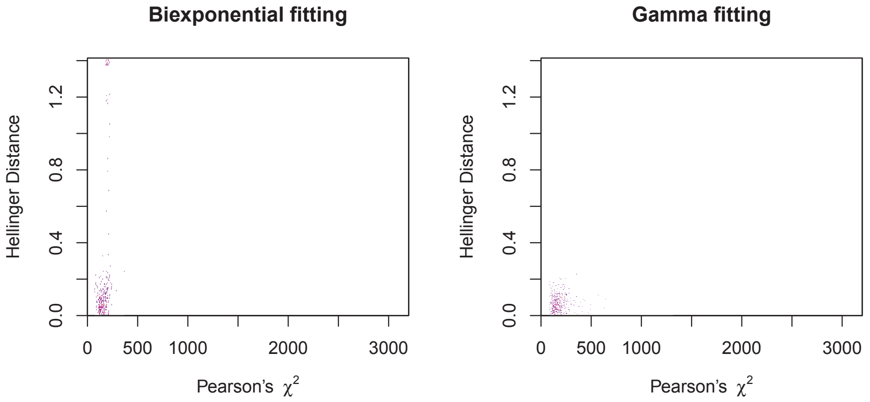

(b) $c = 0.90$, $k = 1.20$

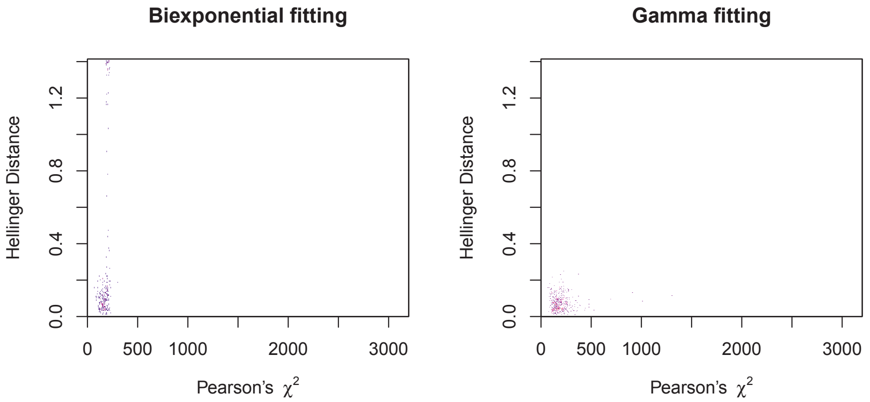

(c) $c = 0.90$, $k = 2.00$

Figure 38: Two-dimensional histogram for Hellinger Distance (vertical axis) and Pearson's χ^2 statistic (horizontal axis) for simulated data, where the plotted values have been aggregated over varying true values of c and τ_2. For each subfigure (a) through (c), fitting the biexponential directly gives the plot on the left, and using gamma conversion method gives the plot on the right. Intensity is graded from blue (lowest) to yellow (highest), white indicating no counts
